# On the potential for mapping apparent neural soma density via a clinically viable diffusion MRI protocol

**DOI:** 10.1016/j.neuroimage.2021.118303

**Published:** 2021-10-01

**Authors:** Noemi G. Gyori, Christopher A. Clark, Daniel C. Alexander, Enrico Kaden

**Affiliations:** a Centre for Medical Image Computing, Department of Computer Science, University College London, London, United Kingdom; b Great Ormond Street Institute of Child Health, University College London, London, United Kingdom

## Abstract

Diffusion MRI is a valuable tool for probing tissue microstructure in the brain noninvasively. Today, model-based techniques are widely available and used for white matter characterisation where their development is relatively mature. Conversely, tissue modelling in grey matter is more challenging, and no generally accepted models exist. With advances in measurement technology and modelling efforts, a clinically viable technique that reveals salient features of grey matter microstructure, such as the density of quasi-spherical cell bodies and quasi-cylindrical cell projections, is an exciting prospect. As a step towards capturing the microscopic architecture of grey matter in clinically feasible settings, this work uses a biophysical model that is designed to disentangle the diffusion signatures of spherical and cylindrical structures in the presence of orientation heterogeneity, and takes advantage of B-tensor encoding measurements, which provide additional sensitivity compared to standard single diffusion encoding sequences. For the fast and robust estimation of microstructural parameters, we leverage recent advances in machine learning and replace conventional fitting techniques with an artificial neural network that fits complex biophysical models within seconds. Our results demonstrate apparent markers of spherical and cylindrical geometries in healthy human subjects, and in particular an increased volume fraction of spherical compartments in grey matter compared to white matter. We evaluate the extent to which spherical and cylindrical geometries may be interpreted as correlates of neural soma and neural projections, respectively, and quantify parameter estimation errors in the presence of various departures from the modelling assumptions. While further work is necessary to translate the ideas presented in this work to the clinic, we suggest that biomarkers focussing on quasi-spherical cellular geometries may be valuable for the enhanced assessment of neurodevelopmental disorders and neurodegenerative diseases.

## Highlights

• B-tensor encoding enables estimation of spherical cellular structures in the brain.
• Spherical compartments may provide markers for apparent neural soma density.
• Model parameters can be estimated in a fast and robust way using deep learning.
• Practical acquisition times are achievable on widely available clinical scanners.

## 1 Introduction

Diffusion MRI enables the non-invasive investigation of tissue microstructure in the human brain (Johansen-Berg, Behrens, 2009, Jones, 2011). This imaging technique has been particularly successful in white matter, where it has improved the characterisation of a range of medical conditions including multiple sclerosis (Bagnato, Franco, Li, Kaden, Ye, Fan, Chen, Alexander, Smith, Dortch, Xu, 2019, Ciccarelli, Werring, Wheeler-Kingshott, Barker, Parker, Thompson, Miller, 2001, Hygino da Cruz, Batista, Domingues, Barkhof, 2011, Schmierer, Wheeler-Kingshott, Boulby, Scaravilli, Altmann, Barker, Tofts, Miller, 2007, Werring, Clark, Barker, Thompson, Miller, 1999), Alzheimer’s disease (Acosta-Cabronero, Nestor, 2014, Kantarci, Murray, Schwarz, Reid, Przybelski, Lesnick, Zuk, Raman, Senjem, Gunter, Boeve, Knopman, Parisi, Petersen, Jack, Dickson, 2017, Schouten, Koini, Vos, Seiler, Rooij, Lechner, Schmidt, Heuvel, Grond, Rombouts, 2017), Huntington’s disease (McColgan, Seunarine, Gregory, Razi, Papoutse, Long, Mills, Johnson, Durr, Roos, Leavitt, Stout, Scahill, Clark, Rees, Tabrizi, Investigators, 2017, Novak, Seunarine, Gibbard, Hobbs, Scahill, Clark, Tabizi, 2014), ischemic stroke (Fung, Roccatagliata, Gonzalez, Schaefer, 2011, Lutsep, Albers, de Crespigny, Kamat, Marks, Moseley, 1997, Warach, Chien, Li, Ronthal, Edelman, 1992), Autism spectrum disorder (Ameis, Lerch, Taylor, Lee, Viviano, Pipitone, Nazeri, Croarkin, Voineskos, Lai, Crosbie, Brian, Soreni, Schachar, Szatmari, Arnold, Anagnostou, 2016, Gibbard, Ren, Seunarine, Clayden, Skuse, Clark, 2013, Gibbard, Ren, Skuse, Clayden, Clark, 2017, Travers, Adluru, Ennis, Tromp, Destiche, Doran, Bigler, Lange, Lainhart, Alexander, 2012) and congenital hypothyroidism (Cooper et al., 2019). It has also enabled mapping fibre connectivity in white matter (Clayden, 2013, Conturo, Lori, Cull, Akbudak, Snyder, Shimony, McKinstry, Burton, Raichle, 1999, Jones, Simmons, Williams, Horsfield, 1999) and can be used to inform neurosurgical planning (Clark, Barrick, Murphy, Bell, 2003, Essayed, Zhang, Unadkat, Rees Cosgrove, Golby, O’Donnell, 2017). Grey matter in comparison has received less attention in the diffusion MRI community, despite its highlighted importance in cognition (Dempster, Norman, Theberge, Densmore, Schaefer, Williamson, 2017, Minatogawa-Chang, Schaufelberger, Ayres, Duran, Gutt, Murray, Rushe, McGuire, Menezes, Scazufca, Busatto, 2009, Zimmerman, Brickman, Paul, Grieve, Tate, Gunstad, Cohen, Aloia, Williams, Clark, Whitford, Gordon, 2006), brain plasticity (Jiang, Yin, Li, Li, Zhao, Evans, Jiang, Wu, Wang, 2015, Tang, Lu, Fan, Yang, Pasner, 2012, Zatorre, Fields, Johansen-Berg, 2012), neurodegeneration (Eshagi, Prados, Brownlee, Altmann, Tur, Cardoso, De Angelis, van de Pavert, Cawley, De Stefano, Stromillo, Battaglini, Ruggieri, Gasperini, Filippi, Rocca, Rovira, Sastre-Garriga, Vrenken, Leurs, Killestein, Pipamer, Enzinger, Ourselin, Gandini Wheeler-Kingshott, Chard, Thompson, Alexander, Barkhof, Ciccarelli, 2018, Gellersen, Guo, O’Callaghan, Tan, Sami, Hornberger, 2017, Vercellino, Masera, Lorenzatti, Condello, Merola, Mattioda, Tribolo, Capello, Mancardi, Mutani, Giordana, Cavalla, 2009, Weston, Simpson, Ryan, Ourselin, Fox, 2015) and neurodevelopment (Adler, Lorio, Jacques, Benova, Gunny, Cross, Baldeweg, Carmichael, 2017, Jernigan, Baare, Stiles, Skak Madsen, 2011, Perani, Tierney, Centeno, Shamshiri, Yaakub, O’Muicheartaigh, Carmichael, Richardson, 2018, Tyborowska, Volman, Niermann, Pouwels, Smeekens, Cillessen, Toni, Roelofs, 2018, Young, Powell, Morgan, Card, Lee, Smith, Sled, Taylor, 2015). There are two key challenges associated with grey matter microstructure imaging that makes it more difficult to deploy in neuroscience research and clinical practice (Assaf, 2019). Firstly, diffusion imaging voxels in clinical settings tend to be large in comparison to cortical thickness, which may lead to substantial partial volume effects with CSF and white matter. Secondly, current biophysical models for brain tissue typically do not consider cell bodies, which tend to be more abundant in grey matter than in white matter, as conventional diffusion MRI measurements have limited sensitivity to different cellular components. In the following, we concentrate on the second challenge and outline recent advances in biophysical modelling, diffusion MR acquisition and computational modelling, which may facilitate the development of microstructural imaging methodology with particular focus on grey matter.

Researchers often use a biophysical model that identifies key cellular structures a priori and approximates these as simple geometrical shapes in order to extract specific information on brain microanatomy efficiently with diffusion MRI (Alexander et al., 2019). In the brain, the cellular structures that are typically considered are thin, quasi-cylindrical projections such as axons, neuronal dendrites and glial processes (Assaf, Basser, 2005, Behrens, Woolrich, Jenkinson, Johansen-Berg, Nunes, Clare, Matthews, Brady, Smith, 2003b, Fieremans, Jensen, Helpern, 2011, Jespersen, Kroenke, Ostergaard, Ackermann, Yablonskiy, 2007, Kaden, Kelm, Carson, Does, Alexander, 2016b, Kaden, Knosche, Anwander, 2007, Kaden, Kruggel, Alexander, 2016a, Lampinen, Szczepankiewicz, Martensson, van Westen, Sundgren, Nilsson, 2017, Zhang, Schneider, Wheeler-Kingshott, Alexander, 2012), which this work collectively refers to as neural projections following Ozarslan et al. (2018). Stanisz et al. (1997) developed a more holistic model based on bovine optic nerve that also considers the diffusion signal from quasi-spherical glial cells. However, this model assumes that orientation dispersion is negligible and hence is not applicable in the brain, which features crossing and fanning fibres. In white matter, models that focus on cylindrical geometries can yield reasonable estimates of the volume fraction and organisation of fibres (Jelescu, Budde, 2017, Panagiotaki, Schneider, Siow, Hall, Lythgoe, Alexander, 2012). In contrast, this is not the case in grey matter, where McKinnon et al. (2017) showed that the diffusion signal attenuation is markedly different from white matter. First, Shemesh and Cohen (2011), Jespersen et al. (2013) linked the decreased microscopic fractional anisotropy in grey matter, measured with double diffusion encoding in ex-vivo pig and monkey brain, to the presence of cell bodies. More recently, Tax et al. (2020) used ultra high magnetic field gradients and isotropic diffusion encoding to detect a spherical compartment with vanishing diffusivity in human grey matter. Meanwhile, Palombo, Ianus, Guerreri, Nunes, Alexander, Shemesh, Zhang, 2020, Palombo, Ianus, Guerreri, Nunes, Alexander, Shemesh, Zhang, 2021, Palombo, Nunes, Alexander, Zhang, Shemesh, 2019, Palombo, Shemesh, Ianus, Alexander, Zhang, 2018 proposed a model with spherical and cylindrical compartments and suggest that the spherical compartment primarily reflects neural soma. Using single diffusion encoding protocols including high b-values, they show maps of spherical and cylindrical component signal fraction estimates in mouse brain, as well as preliminary results in healthy human brain, which reflect contrast in histological images stained for soma and neural projections, respectively.

Fitting complex biophysical models to single diffusion encoding data acquired within practical diffusion times may lead to degeneracy in parameter estimation (Jelescu, Veraart, Fieremans, Novikov, 2016, Kiselev, 2010, Novikov, Veraart, Jelescu, Fieremans, 2018). This problem can be mitigated with more advanced diffusion measurements, such as double diffusion encoding and B-tensor encoding (Coelho, Pozo, Jespersen, Jones, Frangi, 2019, Reisert, Kiselev, Dhital, 2019). Double diffusion encoding plays out two pairs of gradient pulses before signal readout (Cheng, Cory, 1999, Cory, Garroway, Miller, 1990, Mitra, 1995, Shemesh, Ozarslan, Kolmosh, Basser, Cohen, 2010b). By changing the angle between the consecutive gradient pulses, it is possible to be sensitive to both compartment anisotropy (Callaghan, Komlosh, 2002, Jespersen, Lundell, Sonderby, Dyrby, 2013, Komlosh, Horkay, Freidlin, Nevo, Assaf, Basser, 2007, Ozarslan, 2009, Ozarslan, Basser, 2008, Shemesh, Cohen, 2011) and compartment size (Finsterbusch, Koch, 2008, Koch, Finsterbusch, 2008, Komlosh, Ozarslan, Lizak, Horkay, Schram, Shemesh, Cohen, Basser, 2011, Shemesh, Ozarslan, Basser, Cohen, 2010a, Weber, Ziener, Kampf, Herold, Bauer, Jakob, 2009) in the presence of orientation heterogeneity. An alternative strategy is isotropic diffusion encoding introduced by Mori and van Zjil (1995), Wong et al. (1995), where the measurement is sensitised to the diffusion process in all directions equally. Under the multiple gaussian component assumption, isotropic diffusion encoding provides sensitivity to microscopic diffusion tensor magnitude heterogeneity, while factoring out any effects due to microscopic diffusion anisotropy and orientation dispersion (Lasic, Szczepankiewicz, Eriksson, Nilsson, Topgaard, 2014, Szczepankiewicz, Lasic, van Westen, Sundgren, Englund, Westin, Stahlberg, Latt, Topgaard, Nilsson, 2015, Westin, Knutsson, Paternak, Szczepankiewicz, Ozarslan, van Westen, Mattisson, Bogren, O’Donnell, Kubicki, Topgaard, Nilsson, 2016). B-tensor encoding is a measurement strategy that combines different types of diffusion encoding gradient waveforms, such as linear tensor encoding (LTE), which includes conventional single diffusion encoding measurements, and spherical tensor encoding (STE), which is analogous to isotropic diffusion encoding in the absence of confounding time-dependence effects from the diffusion encoding waveforms. Using such measurement combinations, it is possible to disentangle different sources of tissue heterogeneity (Eriksson, Lasic, Nilsson, Westin, Topgaard, 2015, Lasic, Szczepankiewicz, Eriksson, Nilsson, Topgaard, 2014, Szczepankiewicz, Lasic, van Westen, Sundgren, Englund, Westin, Stahlberg, Latt, Topgaard, Nilsson, 2015, Szczepankiewicz, van Westen, Englund, Westin, Stahlberg, Latt, Sundgren, Nilsson, 2016, Westin, Knutsson, Paternak, Szczepankiewicz, Ozarslan, van Westen, Mattisson, Bogren, O’Donnell, Kubicki, Topgaard, Nilsson, 2016).

Traditional fitting techniques, when used with complex non-linear models, are often time-consuming and prone to estimation errors due to local minima, which prohibits their routine use. Recent developments in computational modelling have inspired a number of different machine learning approaches that aim to improve parameter estimation by avoiding local minima and speeding up computation. For example, using Monte Carlo simulations, Nilsson et al. (2010) created a library of synthetic signals that can be matched to experimental signals to estimate parameters with less rigid modelling assumptions. More recently, Nedjati-Gilani, Schneider, Hall, Cawley, Hill, Ciccarelli, Drobnjak, Gandini Wheeler-Kingshott, Alexander, 2017, Nedjati-Gilani, Schneider, Hall, Wheeler-Kingshott, Alexander, Hill et al. (2021) used a random forest regressor to estimate rotationally invariant parameters. Meanwhile, Golkov et al. (2016) showed that deep learning can be used to dramatically reduce scan times and to streamline data processing. Such computational approaches may facilitate fast and accurate parameter estimation in more complex models.

The aim of this work is to disentangle quasi-spherical and quasi-cylindrical structures in-vivo in the human brain in a step towards mapping differently shaped cellular components under clinically feasible conditions. To achieve this, we leverage B-tensor encoding measurements (Szczepankiewicz, Sjolund, Stahlberg, Latt, Nilsson, 2019b, Topgaard, 2017, Westin, Knutsson, Paternak, Szczepankiewicz, Ozarslan, van Westen, Mattisson, Bogren, O’Donnell, Kubicki, Topgaard, Nilsson, 2016) and use the optimisation framework by Sjolund et al. (2015), Szczepankiewicz et al. (2019a) to design a protocol that has sufficient sensitivity to map spherical and cylindrical compartments. We employ a biophysical model of neural tissue, similar to previous work (Palombo, Ianus, Guerreri, Nunes, Alexander, Shemesh, Zhang, 2020, Stanisz, Szafer, Wright, Henkelman, 1997), which consists of spherical and cylindrical geometries and captures the apparent density and diffusivity of these model compartments. To estimate the model parameters, we develop an artificial neural network that reduces computation times and improves stability dramatically over traditional voxel-by-voxel optimisation procedures. We demonstrate with simulations that the artificial neural network estimates the volume fractions and diffusivities of spherical and cylindrical compartments accurately in idealised situations, and we quantify estimation errors as the signal generation process departs from the assumed model. We present maps of the modelled geometries in healthy subjects and consider the interpretation of the spherical and cylindrical components in terms of biological entities such as neural soma, including neuronal and glial cell bodies, and neural projections, including axons, neuronal dendrites and glial processes. The authors have presented preliminary results in Gyori et al. (2019).

## 2 Theory and methods

### 2.1 B-tensor encoding

MRI measurements can be sensitised to the diffusive motion of molecules in a sample. This is achieved by diffusion encoding magnetic field gradients $G\left(t\right)={\left[G_{x}\left(t\right),G_{y}\left(t\right),G_{z}\left(t\right)\right]}^{T}$ that are applied at time t and satisfy $\int_{0}^{\text{TE}}G\left(t\right)dt=0,$ ignoring any radiofrequency (RF) pulses, where TE denotes the echo time. The diffusion-weighted experiment may be summarised by the b-tensor

(1) $$b=\int_{0}^{\mathrm{TE}}q\left(t^{\prime }\right)q^{T}\left(t^{\prime }\right)dt^{\prime }$$

where

(2) $$q\left(t^{\prime }\right)=\gamma \int_{0}^{t^{\prime }}G\left(t^{\prime \prime }\right)dt^{\prime \prime }$$

and γ is the gyromagnetic ratio. The b-tensor (Westin et al., 2014), traditionally referred to as the b-matrix (Basser et al., 1994), is a symmetric 3×3 matrix. Conventionally, gradients are applied in only one direction per readout period, and the b-tensor can be decomposed into the magnitude of diffusion weighting given by the trace Tr(b), also known as the b-value (Le Bihan et al., 1986), and the direction of the gradients. Such measurements are referred to as linear tensor encoding (LTE) (Westin et al., 2016).

In more recent work, trapezoidal pulses have been replaced by free gradient waveforms to make measurements more efficient and to allow for different b-tensor shapes (Eriksson, Lasic, Nilsson, Westin, Topgaard, 2015, Eriksson, Lasic, Topgaard, 2013, Lasic, Szczepankiewicz, Eriksson, Nilsson, Topgaard, 2014, Szczepankiewicz, Sjolund, Stahlberg, Latt, Nilsson, 2019b, Topgaard, 2017, Westin, Knutsson, Paternak, Szczepankiewicz, Ozarslan, van Westen, Mattisson, Bogren, O’Donnell, Kubicki, Topgaard, Nilsson, 2016). In this case, the b-value and gradient direction representation of diffusion weighting is no longer sufficient and the shape of the b-tensor must also be taken into account. For axially symmetric b-tensors, such as LTE and STE, **b** can be expressed in terms of the traditional b-value (b) and gradient direction (n), and additionally the b-tensor shape $b_{\Delta }$ (Eriksson et al., 2015):

(3) $$b=b_{\Delta }b{\mathrm{nn}}^{T}+\frac{b}{3}\left(1-b_{\Delta }\right)I$$

where I is a 3×3 identity matrix, and $b_{\Delta }\in \left[-1/2,1\right]$.

**Fig. 1:**
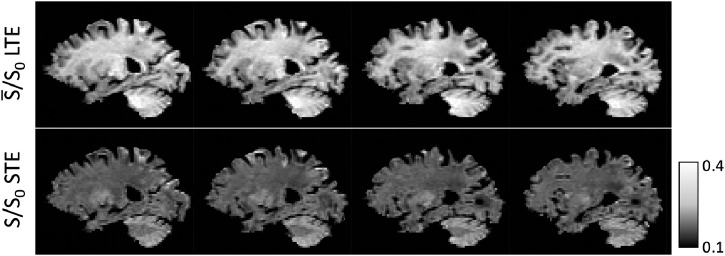
T2-normalised signal at $b=2000\;s/{\text{mm}}^{2}$ using linear tensor encoding (LTE) averaged over gradient directions (top), and spherical tensor encoding (STE) (bottom). In LTE, the diffusion signal is higher in white matter and lower in grey matter, whereas in STE the signal is higher in grey matter and lower in white matter. This contrast inversion effect is especially apparent in the cerebellum.

By making measurements with different $b_{\Delta }$ in addition to different b-values and different gradient directions, more detailed information of the underlying tissue structure can be obtained and different signal contributors can be disentangled. This work focuses on two b-tensor shapes: linear tensor encoding (LTE) where $b_{\Delta }=1,$ and isotropic diffusion encoding, here referred to as spherical tensor encoding (STE) where $b_{\Delta }=0$ and b=diag(b/3,b/3,b/3) (Eriksson et al., 2015). These two b-tensor shape measurements recover strikingly different contrast in the brain (Fig. 1), which motivates the use of the biophysical model discussed in the next section.

### 2.2 Biophysical model

**Fig. 2:**
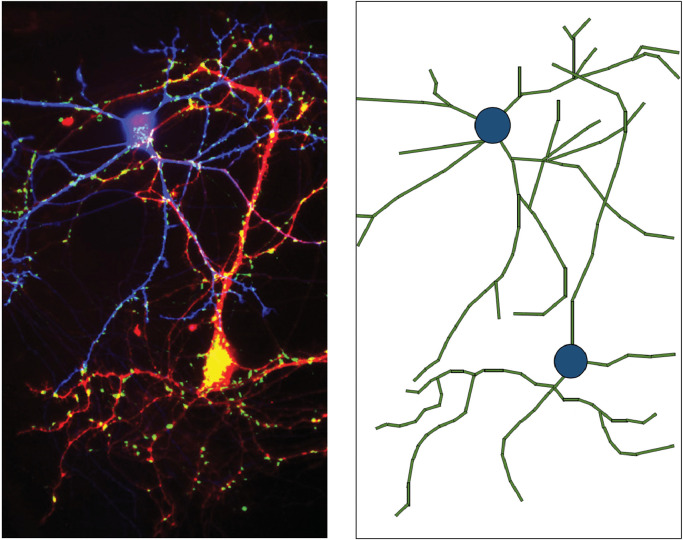
Left: neuron pair under fluorescence microscope. Image credit: Wierda and Sorensen (2014), “Innervation by a GABAergic neuron depresses spontaneous release in glutamatergic neurons and unveils the clamping phenotype of synaptotagmin-1” by K.D.B. Wierda and J.B Sorensen, 2014, J. Neurosci., 34(6), 2100-2110, (DOI:https://doi.org/10.1523/JNEUROSCI.3934-13.2014). CC BY-NC-SA 3.0. Right: schematic representation of neural cell structure. Cell bodies are approximated by spherical geometries (blue) and cellular projections are approximated by cylindrical segments (green). Spatial information of cellular structures within a voxel is not encoded in the MRI measurement. (For interpretation of the references to colour in this figure legend, the reader is referred to the web version of this article.)

In order to disentangle different cellular geometries in the brain, we use a biophysical model similar to previous work (Palombo, Ianus, Guerreri, Nunes, Alexander, Shemesh, Zhang, 2020, Stanisz, Szafer, Wright, Henkelman, 1997), consisting of two different cellular structures as well as the surrounding extra-cellular volume. Firstly, we consider quasi-cylindrical geometries such as axons, neuronal dendrites and glial processes, which we refer to collectively as neural projections. Secondly, we consider quasi-spherical structures such as neuronal and glial cell bodies, which we refer to as neural soma. We highlight that other entities in tissue may also have quasi-spherical shapes, such as vacuoles and large synaptic boutons, but capturing the diffusion signature of these structures is beyond the scope of this work. We assume that T2 relaxation is comparable inside cellular structures and in the extra-cellular volume (Clark, Le Bihan, 2000, Niendorf, Dijkhuizen, Norris, van Lookeren Campagne, Nicolay, 1996). Thus, brain tissue is modelled to consist of three distinct components, such that $v_{\text{cyl}}+v_{\text{sph}}+v_{\text{ext}}=1$ where $v_{i}$ represents the volume fraction of component i. Fig. 2 shows a schematic representation of grey matter morphology composed of quasi-spherical cell bodies and quasi-cylindrical segments of neural projections.

To relate the diffusion signal to cellular structures in tissue, we first consider a microscopic environment of brain tissue that a water molecule explores during the MRI experiment, which is typically in the range of a few micrometers, has a simple architecture with negligible orientation dispersion, and is also referred to as a microdomain (Eriksson, Lasic, Topgaard, 2013, Kaden, Kruggel, Alexander, 2016a). We use a first order approximation with respect to the b-value to model the microscopic signal, whereby diffusion within a microdomain is assumed to be Gaussian and can be described by an axially symmetric tensor, D, with orientation ϕ. Diffusion within cylindrical structures, $D_{\text{cyl}},$ is characterised by two unique eigenvalues parallel and perpendicular to the long axis ($\lambda_{∥}^{\text{cyl}},\lambda_{⊥}^{\text{cyl}}$). Based on results in Drobnjak et al. (2016) we assume that, for the present experimental conditions, diffusivity perpendicular to cylindrical geometries is negligible ($\lambda_{⊥}^{\text{cyl}}=0$) and hence $D_{\text{cyl}}=\text{diag}\left(\lambda_{\text{cyl}},0,0\right)$ in its principal axis system. In spherical cellular structures diffusion is isotropic and so $D_{\text{sph}}=\text{diag}\left(\lambda_{\text{sph}},\lambda_{\text{sph}},\lambda_{\text{sph}}\right)$. We assume that parallel diffusivity in cylindrical structures equals the intrinsic neural diffusivity λ (i.e. $\lambda =\lambda_{\text{cyl}}$) and that $\lambda_{\text{sph}}\leq \lambda$ due to confinement within the cellular compartment. In extra-cellular space, diffusion is hindered by both cylindrical and spherical cellular structures with $D_{\text{ext}}=\text{diag}\left(\lambda_{\text{ext}}^{∥},\lambda_{\text{ext}}^{⊥},\lambda_{\text{ext}}^{⊥}\right)$ in its principal axis system, and we use a tortuosity approximation developed by Stanisz et al. (1997) to determine the resulting microscopic diffusivities. Assuming that the free path of water molecules is longer than the size of cellular compartments and that diffusion is only hindered by spherical and cylindrical structures, $\lambda_{\text{ext}}^{∥}$ and $\lambda_{\text{ext}}^{⊥}$ take the form

(4) $$\lambda_{∥}^{\text{ext}}=\lambda v_{\text{ext}}^{\frac{1}{2}v_{\text{sph}}/\left(v_{\text{sph}}+v_{\text{cyl}}\right)}$$

and

(5) $$\lambda_{⊥}^{\text{ext}}=\lambda v_{\text{ext}}^{\left(\frac{1}{2}v_{\text{sph}}+v_{\text{cyl}}\right)/\left(v_{\text{sph}}+v_{\text{cyl}}\right)}.$$

Overall, the model assumes that the diffusion signal from tissue is composed of multiple Gaussian components and that diffusivities are not dependent on the temporal profile of the diffusion weighting gradient waveforms. So far, diffusivity of the microdomains are modelled in their principal axis system, and their orientation ϕ with respect to the diffusion weighting is ignored. The orientation of microdomains is incorporated into the model using a rotation of the diffusion tensor by R(ϕ) such that $D_{i}\left(\phi \right)=R\left(\phi \right)D_{i}R{\left(\phi \right)}^{T}$ with i∈{cyl,sph,ext}. For the present model, the resulting T2-normalised diffusion signal from each microdomain, h, is given by

(6) $$h\left(b,\phi \right)=\sum_{i}v_{i}\exp\left(-b:D_{i}\left(\phi \right)\right)$$

where $b:D_{i}\left(\phi \right)$ refers to the double inner product of b and $D_{i}\left(\phi \right),$ and i∈{cyl,sph,ext}. The macroscopic signal measured for a voxel can be described by the sum of the signals from each contributing microdomain

(7) $$\frac{S(b)}{S_{0}}=\int h\left(b,\phi \right)P\left(\phi \right)d\phi$$

where P(ϕ) is the orientation distribution of microdomains.

### 2.3 Disentangling orientation dispersion and microstructure

In the model described above (Eqs. (6) and (7)) the diffusion signal is a function of both the diffusion properties of the microdomains and the orientation distribution of cylindrical compartments. In order to characterise tissue microanatomy without the confounding effect of orientation distribution, it is necessary to disentangle microstructural and orientation effects. This can be achieved by rotating the gradient waveforms and taking the powder average of the signals, which is invariant with respect to the orientation distribution (Callaghan, Soderman, 1983, Eriksson, Lasic, Nilsson, Westin, Topgaard, 2015, Kaden, Kruggel, Alexander, 2016a). Thus, the powder-averaged signal, $\bar{S},$ is equal to the powder-average $\bar{h}$ of the microdomain signal in Eq. (6):

(8) $$\frac{\bar{S}\left(b,b_{\Delta }\right)}{S_{0}}=\bar{h}\left(b,b_{\Delta }\right)$$

where

(9) $$\bar{h}\left(b,b_{\Delta }\right)\;=\;\sum_{i}v_{i}\frac{\sqrt{\pi }\text{erf}(\sqrt{bb_{\Delta }\left(\lambda_{∥}^{i}-\lambda_{⊥}^{i}\right)})}{2\sqrt{bb_{\Delta }\left(\lambda_{∥}^{i}-\lambda_{⊥}^{i}\right)}}\exp(\;-b[\frac{1-b_{\Delta }}{3}\lambda_{∥}^{i}+\frac{2+b_{\Delta }}{3}\lambda_{⊥}^{i}])$$

where erf is the error function, $\lim_{x\to 0}\text{erf}\left(x\right)/x=2/\sqrt{\pi },$ and $\lambda_{∥}^{i}$ and $\lambda_{⊥}^{i}$ are the parallel and perpendicular diffusivities of spherical, cylindrical and extra-cellular compartments. The derivation of the general form of the powder-averaged signal for b-tensor diffusion encoding can be found in Eriksson et al. (2015). Once orientation effects are eliminated with the powder average, the biophysical model we use in this work has four independent model parameters: $v_{\text{cyl}},\;v_{\text{sph}},\;\lambda_{\text{cyl}}$ and $\lambda_{\text{sph}}$. From these, we infer the volume fraction and diffusivity of extra-cellular space using Eqs. (4) and (5).

We keep the model as simple as possible aiming to capture the primary effects. This both stabilises parameter estimation and simplifies reasoning about departures from the model that occur in real biological tissue, both healthy and pathological. To aid the latter, we also introduce later a range of simulation experiments that quantify key departures from the model that we are likely to encounter in real tissue.

### 2.4 Experiment design

In this work we used a combination of linear tensor encoding (LTE) and spherical tensor encoding (STE). To maximise the achievable b-value for a given echo time, we designed the STE waveform using the optimisation framework in Sjolund et al. (2015). In the optimal scenario, diffusion encoding waveforms are asymmetric around the 180° refocusing RF pulse to allow time for the EPI readout following the diffusion gradients. Asymmetric waveforms introduce unwanted Maxwell terms, and to reduce these we used methods described in Szczepankiewicz et al. (2019a). In this work, an STE waveform was optimised for maximum gradient strength of 70 mT/m, maximum slew rate of 100 T/m/s. To enable safe waveform rotation, the optimisation used Euclidean norm such that the norm of the co-ordinate components of the waveforms, $\sqrt{G_{x}^{2}+G_{y}^{2}+G_{z}^{2}},$ did not exceed the chosen maximum gradient strength (Sjolund et al., 2015). With a waveform duration of 77 ms, the maximum b-value is 2000 $s/{\text{mm}}^{2}$ for STE. LTE was designed using symmetric trapezoidal pulses, for which $b=5000\;s/{\text{mm}}^{2}$ requires a gradient strength of less than 50 mT/m for a waveform duration of 77 ms. The waveforms are shown in Fig. S7 (Supplementary Material).

After informed written consent was obtained, six healthy volunteers (4 females, 2 males, ages = 26.3±1.5 years) were scanned on a 3T Siemens Prisma scanner using a 64-channel head coil. Ethical approval for the study was obtained from the UCL Research Ethics Committee. We used a prototype pulse sequence based on the product diffusion-weighted spin-echo EPI sequence that facilitates execution of free gradient waveforms (Szczepankiewicz et al., 2019c). B-values of [500, 1000, 1500, 2000] $s/{\text{mm}}^{2}$ were measured for STE, and b-values of [1000, 2000, 3500, 5000] $s/{\text{mm}}^{2}$ were measured for LTE. The higher b-values obtained for LTE are beneficial for reliably estimating the four independent parameters of the model used in this work. For both LTE and STE the waveform was rotated in 128 uniformly distributed gradient directions over the four b-shells (Caruyer et al., 2013). 12 b0 images were measured, with an additional b0 image with reversed phase encoding. We measured isotropic 2 mm voxels with acquisition matrix 128×128×70, partial Fourier imaging 0.75, field of view $256\times 256\times 140\;{\text{mm}}^{3},$ TE = 94 ms and TR = 9.2 s, and GRAPPA parallel imaging with acceleration factor 2. The total acquisition time with these parameters was 43 min, and the resulting SNR in the diffusion measurements was approximately 25. In addition, a 3D T1-weighted MPRAGE with 1 mm isotropic resolution was acquired.

### 2.5 Data pre-processing

Gibbs ringing artefacts were removed using the method described in Kellner et al. (2016). Data was further pre-processed using the FSL toolbox (Smith et al., 2004). Firstly, the susceptibility-induced off-resonance field was estimated by using two b0 measurements with reversed phase encoding polarities (Andersson et al., 2003). Secondly, eddy currents and subject movement were corrected for using methods described in Andersson and Sotiropoulos (2016). LTE and STE data were pre-processed separately, but subject motion was corrected relative to the same b0 image to ensure that LTE and STE were co-registered. A mask to remove non-brain regions from the images was also created (Smith, 2002). Finally, we adjusted for Rician noise bias in our in-vivo maps using the method described in Kaden et al. (2016a). In order to compare results in different brain regions, the T1-weighted data was segmented using FreeSurfer (Fischl, 2012) and then co-registered to the diffusion data.

### 2.6 Deep-learning model fitting

For fast and robust estimation of model parameters, we developed a neural network consisting of three fully connected layers with rectified linear unit activation functions (Goodfellow et al., 2016). The three-layer neural network is able to capture the complexity of the biophysical model sufficiently well (Supplementary Material, Fig. S2) and gives similar results to traditional model fitting techniques (Supplementary Material, Figs. S3 and S4). Our implementation is based on the PyTorch deep-learning platform (https://pytorch.org).

The network inputs are the T2-normalised powder-averaged diffusion signals and the standard deviation of the Gaussian noise in these signals. The rationale is (i) to factor out unwanted orientation effects, (ii) to make the network independent of the gradient directions and hence generalisable as long as the b-values, the type of encoding, e.g. LTE or STE, and the relative number of gradient directions per b-value are unchanged, (iii) to cope with varying noise levels across the image when surface coils are used, and (iv) to enable the usage of the same network for a wide range of protocols with different echo times, voxel sizes and signal readouts that all influence the signal-to-noise ratio. The network outputs are

$$\begin{array}{ccc} z_{1} & = & \mathrm{logit}(v_{\text{cyl}}+v_{\text{sph}}),\\ z_{2} & = & \mathrm{logit}(v_{\text{cyl}}/\left(v_{\text{cyl}}+v_{\text{sph}}\right)),\\ z_{3} & = & \mathrm{logit}(\lambda_{\text{cyl}}/\lambda_{\text{free}}) \end{array}$$

and

$$z_{4}=\mathrm{logit}\left(\lambda_{\text{sph}}/\lambda_{\text{cyl}}\right),$$

where logit(p)=log(p)−log(1−p) denotes the logit function and $\lambda_{\text{free}}=3.0\;\mu m^{2}/\text{ms}$ is the diffusivity of free water at body temperature of 37°C. This transformation improves the training performance and guarantees by design that the estimated parameters are in their biophysically plausible range, i.e. $v_{i}\in \left[0,1\right],\sum_{i}v_{i}=1$ for $i\in \left\{\text{cyl},\text{sph},\text{ext}\right\},\lambda_{\text{cyl}}\in \left[0,\lambda_{\text{free}}\right]$ and $\lambda_{\text{sph}}\in \left[0,\lambda_{\text{cyl}}\right]$.

We trained the neural network using a synthetic data set generated with the forward model formulated in Eqs. (8) and (9). To sample the model parameters as uniformly as possible given the model constraints, both the volume fractions and the diffusivities were drawn from a uniform distribution on a 2 -simplex. The logarithm of the standard deviation log(σ) was sampled from a uniform distribution on [log(0.01),log(1)], which is a noninformative, scale-invariant prior distribution (Robert, 2007) that covers signal-to-noise ratios between 1 and 100. A total of $2^{20}$ measurements were synthesised, of which 75% were used to train the network and 25% to validate its performance.

The neural network was trained batch-wise with 100,000 epochs using a mean-squared-error loss criterion and a stochastic gradient descent optimiser with a learning rate of 0.001 and momentum of 0.9, which produced good convergence results. To avoid overfitting, we added Gaussian noise with variable standard deviation σ to the simulated signals for each batch and epoch independently. Eventually, the trained network was used to map the LTE and STE diffusion measurements to the four model parameters $v_{\text{cyl}},\;v_{\text{sph}},\;\lambda_{\text{cyl}}$ and $\lambda_{\text{sph}}$ for each voxel individually without spatial regularisation or prior denoising, which can be achieved within seconds either on a CPU or GPU.

## 3 Results

In this section we present our results in four key areas. First, we use simulations to test the accuracy of parameter estimation under different experimental conditions, such as different SNR and different number of gradient directions, for the idealised case where all of the model assumptions hold. In Section 3.2, we use simulations to test the accuracy of parameter estimation in the presence of various departures from the biophysical model, such as signal contamination from CSF, differing compartmental T2-relaxation times, non-negligible perpendicular diffusivity in cylindrical compartments, presence of myelin water, deviations from the tortuosity model for extra-cellular diffusivity, and different distributions of spherical diffusivities. In Section 3.3, we fit the model to the in-vivo experimental data and show maps of the model parameters, $v_{\text{sph}},\;v_{\text{cyl}},\;\lambda_{\text{cyl}},\;\lambda_{\text{sph}}$. Finally, in Section 3.4 we probe the feasibility of shorter acquisition times by fitting the model to smaller data sets.

### 3.1 Performance of parameter estimation in the idealised model

To determine whether the developed artificial neural network can accurately recover model parameters, it was first tested on data sets that were synthesised independently of the training data. The test data sets in this case were generated with the same biophysical model (Eqs. (8) and (9)) as the training data. For each dataset, the model parameters were fixed and the diffusion signal was simulated using 10,000 Gaussian noise instances at SNR = 25, matching the SNR of the in-vivo data set used in this work. Compartment diffusivities were varied between [0.5, 3.0] $\mu m^{2}/\text{ms}$ at increments of 0.5 $\mu m^{2}/\text{ms},$ whereas compartment volume fractions were varied between [0, 1] at increments of 0.05, such that all model constraints were satisfied.

**Fig. 3:**
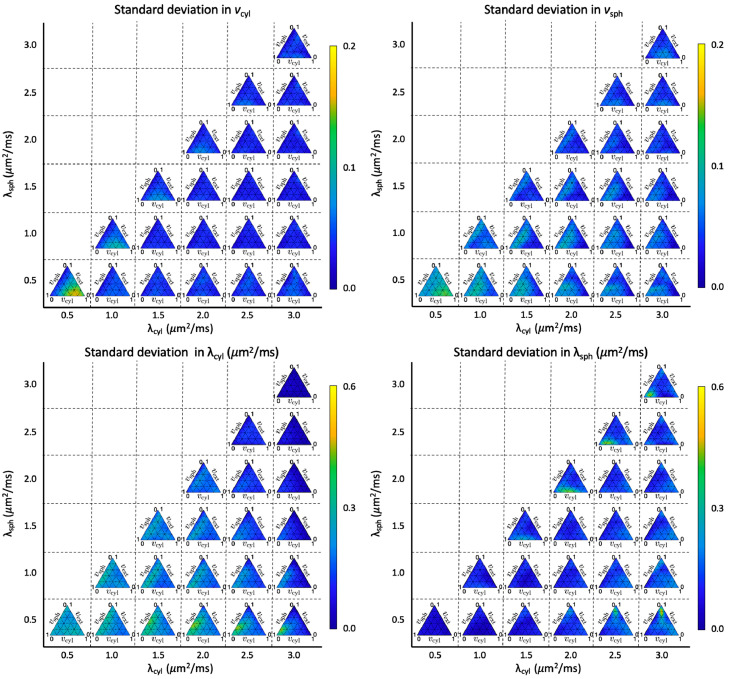
Standard deviation in estimated parameters for different regions of the plausible parameter space. Each simplex is composed of 231 different combinations of compartment volume fractions and fixed compartment diffusivities. For each point, 10,000 noise instances were synthesised using Gaussian noise with SNR = 25. Regions where parameters are estimated with high precision appear blue, and regions where parameters are estimated with low precision appear yellow, as shown by the colour bar. (For interpretation of the references to colour in this figure legend, the reader is referred to the web version of this article.)

**Fig. 4:**
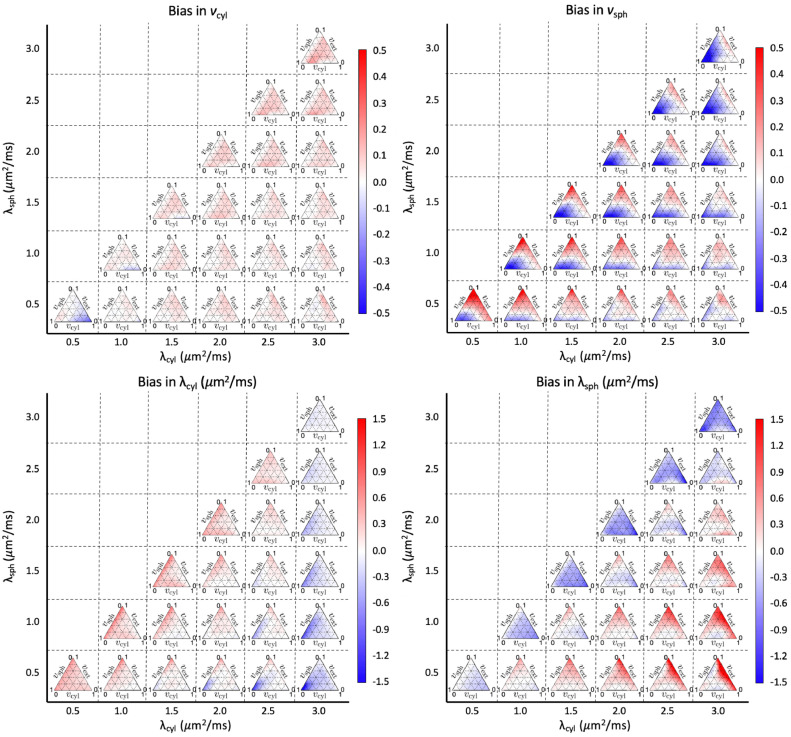
Bias in estimated parameters for different regions of the plausible parameter space. Each simplex is composed of 231 different combinations of compartment volume fractions and fixed compartment diffusivities. For each point, 10,000 noise instances were synthesised using Gaussian noise with SNR = 25. Red regions show where parameter estimates tend to be overestimated, whereas blue regions show where parameter estimates tend to be underestimated. In human brain tissue, we might expect $\lambda_{\text{cyl}}$ to be in the range of 2 - 2.5 $\mu m^{2}/\text{ms},$ and for $\lambda_{\text{sph}}$ to be low. (For interpretation of the references to colour in this figure legend, the reader is referred to the web version of this article.)

Each test set was evaluated by the network described in Section 2.6, and the estimated parameters were compared to the ground truth values. Figs. 3 and 4 show the standard deviation and bias, respectively, obtained for each of the parameters. Fig. 3 shows that for both $v_{\text{cyl}}$ and $v_{\text{sph}},$ standard deviation in estimates is particularly high when both compartment diffusivities are low and as $v_{\text{cyl}}\to 1$. For $\lambda_{\text{cyl}},$ precision tends to be low when $v_{\text{cyl}}\to 0$ and when compartment diffusivities are low. Meanwhile, for $\lambda_{\text{sph}},$ precision is low when $v_{\text{ext}}\to 1,\;\lambda_{\text{sph}}$ is low and $\lambda_{\text{cyl}}\to 3\;\mu m^{2}/\text{ms},$ or when $v_{\text{sph}}\to 1$ and $\lambda_{\text{sph}}\to \lambda_{\text{cyl}}$.

**Fig. 5:**
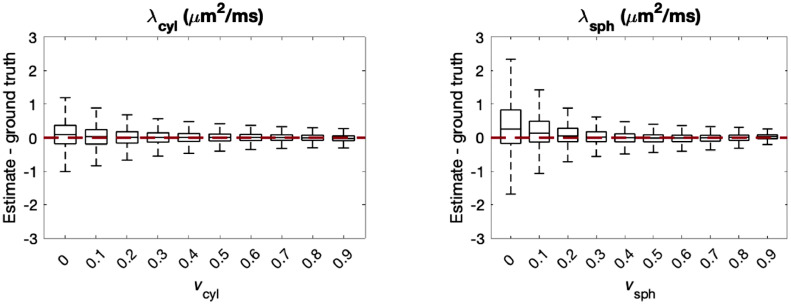
Estimation errors in cylindrical and spherical compartment diffusivities at different volume fractions of the corresponding compartment. For each box, the relevant volume fraction was fixed, while for the remaining parameters 107,520 samples were uniformly drawn from the plausible parameter space. Boxes show the range between the 25th and 75th percentiles with whiskers extending 1.5 times the interquartile range. The red line shows where the estimates equal the ground truth.

Fig. 4 reveals that for $v_{\text{sph}},$ measurements tend to be biased as $\lambda_{\text{sph}}\to \lambda_{\text{cyl}}$ and as $v_{\text{cyl}}\to 0$. This effect is likely due to an ambiguity in the biophysical model in the absence of cylindrical compartments, which is exacerbated when $\lambda_{\text{sph}}$ is high or close to $\lambda_{\text{cyl}}$. In human brain tissue, previous works (Kaden, Kelm, Carson, Does, Alexander, 2016b, Novikov, Veraart, Jelescu, Fieremans, 2018) found $\lambda_{\text{cyl}}$ to be approximately 2 $\mu m^{2}/\text{ms}$ and higher, whereas we expect $\lambda_{\text{sph}}$ to be low, as this term entangles diffusivity and restriction effects. In regions of low $\lambda_{\text{sph}}$ and high $\lambda_{\text{cyl}}$ bias in $v_{\text{sph}}$ is more moderate. However, as $\lambda_{\text{sph}}\to \lambda_{\text{cyl}},$ estimates in $v_{\text{sph}}$ may be unreliable. Bias in $v_{\text{cyl}}$ tends to be moderate throughout the parameter space. Fig. 4 also reveals biases in the compartment diffusivities when $\lambda_{\text{cyl}}\to 3\;\mu m^{2}/\text{ms}$ and $\lambda_{\text{sph}}$ is low. In these regions, bias in $\lambda_{\text{cyl}}$ is particularly pronounced as $v_{\text{cyl}}\to 0,$ whereas bias in $\lambda_{\text{sph}}$ is particularly pronounced as $v_{\text{sph}}\to 0$. This shows that it may be difficult to estimate compartment diffusivities accurately when the corresponding compartment volume fraction is low. We further demonstrate this effect with box and whisker plots in Fig. 5. For each box, we fixed either $v_{\text{cyl}}$ or $v_{\text{sph}},$ and sampled the other parameters uniformly. On average, errors in the compartment diffusivities increase as the corresponding volume fraction decreases. These results suggest that estimates of $\lambda_{\text{sph}}$ may be unreliable in white matter and estimates of $\lambda_{\text{cyl}}$ may be unreliable in grey matter.

#### 3.1.1 Different noise levels

To test the behaviour of the parameter estimation at different values of SNR, data was synthesised with different noise levels, but still using the idealised case of the biophysical model. Here we pool error statistics over all parameter combinations used in Figs. 3 and 4 rather than map them for each combination. As before, we use the same neural network described in Section 2.6 to evaluate each noisy test data set. We compare the resulting estimates to the ground truth using box plots, where boxes show the range between the 25th and 75th percentiles and whiskers span 1.5 times the interquartile range. As parameters are drawn uniformly from all possible parameter combinations, the box plots may give a pessimistic view of the estimation accuracy and precision, as they include parameter combinations that do not typically occur in the brain, but for which estimation bias is high, such as in the corners where $v_{\text{sph}}\to 1$ or $v_{\text{ext}}\to 1$.

**Fig. 6:**
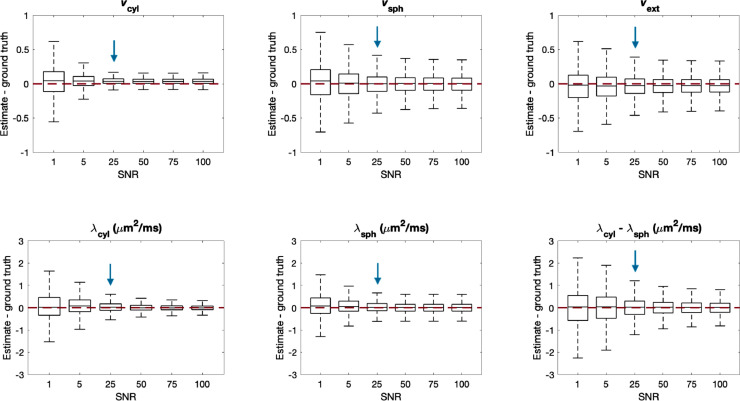
Difference between parameter estimates and ground truth for different values of SNR in the test data for 128 gradient directions for STE and LTE each, as in the in-vivo protocol. For each box, 107,520 sets of parameters were uniformly drawn from the entire plausible parameter space. Boxes show the range between the 25th and 75th percentiles with whiskers extending 1.5 times the interquartile range. The red line shows where the estimates equal the ground truth, and the blue arrow shows the SNR corresponding to the in-vivo data in this study. The figure demonstrates that parameters can be estimated from data with SNR=25 with reasonable accuracy. (For interpretation of the references to colour in this figure legend, the reader is referred to the web version of this article.)

Fig. 6 shows box plots of the difference between the estimated and ground truth parameters for SNR∈[1,5,25,50,75,100]. The figure demonstrates that higher SNR tends to improve both the accuracy and precision of parameter estimation. However, only slight improvements may be seen for SNR>50. The reason for this is that the three-layer neural network has limited capacity and further layers should be added to observe increased accuracy for higher SNR, as Supplementary Fig. S2 suggests.

#### 3.1.2 Different numbers of gradient directions

The performance of parameter estimation via the artificial neural network was also tested for different numbers of gradient directions. The in-vivo data was acquired with 128 gradient directions over four b-shells for both the STE and LTE acquisitions. Here, data with different numbers of gradient directions, but constant SNR=25 was synthesised. As in the previous section, data was synthesised using 107,520 different parameter combinations sampled uniformly from the entire plausible parameter space.

**Fig. 7:**
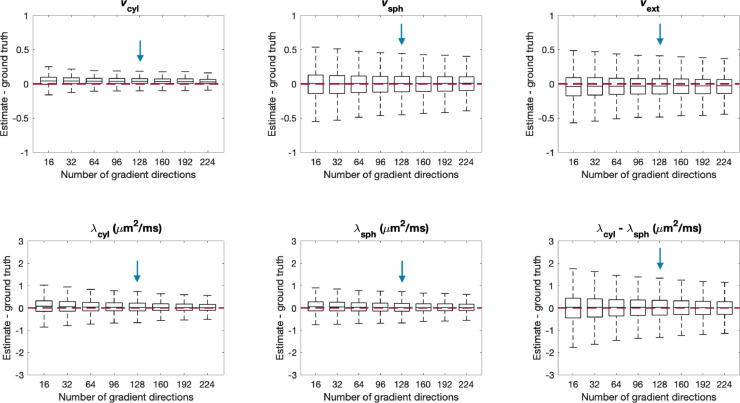
Difference between parameter estimates and ground truth for different numbers of gradient directions for data with SNR = 25. For each box, 107,520 sets of parameters were uniformly drawn from the entire plausible parameter space. The red line shows where the estimates equal the ground truth, and the blue arrow shows the number of gradient directions that were acquired in this study. The figure indicates that parameter estimation is reasonably accurate with 128 gradient directions for LTE and STE each, and could be reduced without major penalties in accuracy. (For interpretation of the references to colour in this figure legend, the reader is referred to the web version of this article.)

In Fig. 7, we show box plots of the difference between estimated and ground truth parameters for different numbers of gradient directions in the range of [16,224] directions. Fig. 7 shows that the number of gradient directions used in this work yields reasonable accuracy and that the number of gradient directions can potentially be reduced without incurring major penalties in the overall accuracy of parameter estimation.

### 3.2 Accuracy of parameter estimation when modelling assumptions are violated

#### 3.2.1 CSF contamination

To investigate the effect of CSF on our parameter estimates, we synthesised data that contains all of our modelled compartments as well as a fourth CSF compartment. As before, samples were drawn uniformly from the entire plausible parameter range to ensure all parameter combinations were represented in the test data. We accounted for differing T2-relaxation between tissue and CSF explicitly and generated the data assuming that $T2_{\text{CSF}}=2\;s$ and $T2_{\text{tissue}}=0.1\;s,$ as suggested by Spijkerman et al. (2018). We fixed CSF diffusivity to 3 $\mu m^{2}/\text{ms}$ and accounted for CSF volume fraction such that $v_{\text{cyl}}+v_{\text{sph}}+v_{\text{ext}}+v_{\text{CSF}}=1$. We simulated signals with the same protocol with which the in-vivo data was collected, and added noise with SNR = 25 which reflects the noise in our in-vivo data. With this set-up we generated 9 additional data sets with varying $v_{\text{CSF}},$ from which we estimated our original model parameters with the same artificial neural network as described in the Methods section. We expect the model to accommodate the CSF signal in the extra-cellular compartment and thus use $v_{\text{ext}}+v_{\text{CSF}}$ as the gold standard for the extra-cellular space volume fraction.

**Fig. 8:**
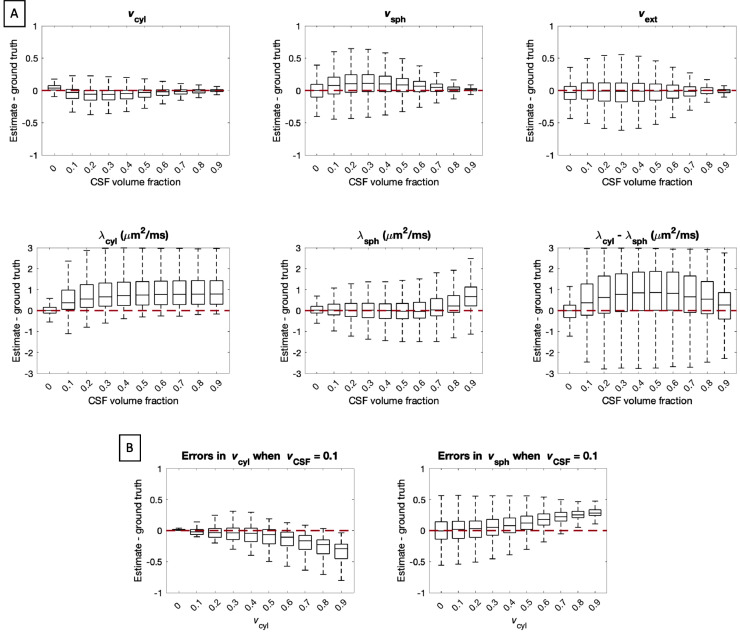
Panel (A): Box plots showing the estimation errors of all parameters when a CSF compartment is introduced with different T2 relative to tissue. For each box, 107,520 sets of parameters were uniformly drawn from the entire plausible parameter space. The precision of all parameter estimates is adversely affected by partial volume with CSF. In the range $0.1<v_{\text{CSF}}<0.7,$ CSF contamination manifests as a bias in $v_{\text{cyl}}$ and $v_{\text{sph}}$. Panel (B): Box plots showing the estimation errors for $v_{\text{cyl}}$ and $v_{\text{sph}}$ when $v_{\text{CSF}}$ is kept constant at 0.1, which is representative for CSF partial volume in human grey matter (Bender, Klose, 2009, Ernst, Kreis, Ross, 1993), and $v_{\text{cyl}}$ is varied. For each box, $v_{\text{cyl}}$ was fixed and the remaining parameters were sampled uniformly. The plots demonstrate that the effect of CSF contamination on the compartment volume fractions is stronger when there is a higher volume fraction of cylindrical compartments.

Fig. 8 A shows that CSF adversely affects the precision and accuracy of all parameter estimates. In particular, for $0.1<v_{\text{CSF}}<0.7,$ CSF contamination manifests as a bias in $v_{\text{cyl}}$ and $v_{\text{sph}}$. The model used in this work does not explicitly model free water, but one might expect that the extra-cellular compartment absorbs the CSF contribution to the signal. In the absence of cellular compartments, the extra-cellular water is isotropic, and hence can indeed capture free water. In the presence of cylindrical compartments, the tortuosity approximation dictates that extra-cellular space diffusivity should be anisotropic. Thus, when both CSF and cylindrical compartments are present within a voxel, extra-cellular space is expected to be anisotropic and cannot capture free water. Instead, CSF is attributed to spherical compartments with high apparent diffusivity. This suggests that the bias observed in the presence of CSF contamination is dependent on $v_{\text{cyl}}$. To demonstrate this effect, we created further test data sets, where $v_{\text{CSF}}=0.1,$ which is representative for CSF partial volume in human grey matter (Bender, Klose, 2009, Ernst, Kreis, Ross, 1993), and where $v_{\text{cyl}}$ was varied between 0 and 0.9. Results presented in Fig. 8B show that bias due to CSF contamination is indeed stronger as $v_{\text{cyl}}$ is increased, particularly for estimates of $v_{\text{cyl}}$ and $v_{\text{sph}}$.

#### 3.2.2 Compartmental T2-relaxation times

The biophysical model in this work assumes that T2-relaxation is the same across all tissue compartments. While Clark and Le Bihan (2000) showed that this is a reasonable assumption in the healthy human brain, other studies (McKinnon, Jensen, 2019, Veraart, Novikov, Fieremans, 2018) found differences between intra- and extra-compartmental T2-relaxation times. To investigate this in our case, we synthesised data with the original model compartments, but with different T2 values for intra-cellular compartments (cylindrical and spherical) and extra-cellular space. We fixed intra-cellular T2 to 0.1 s and varied extra-cellular T2 in the range of 0.03-0.1 s motivated by the results found in McKinnon and Jensen (2019); Veraart et al. (2018). As before, data was synthesised using 107,520 different parameter combinations sampled uniformly from the entire plausible parameter space.

**Fig. 9:**
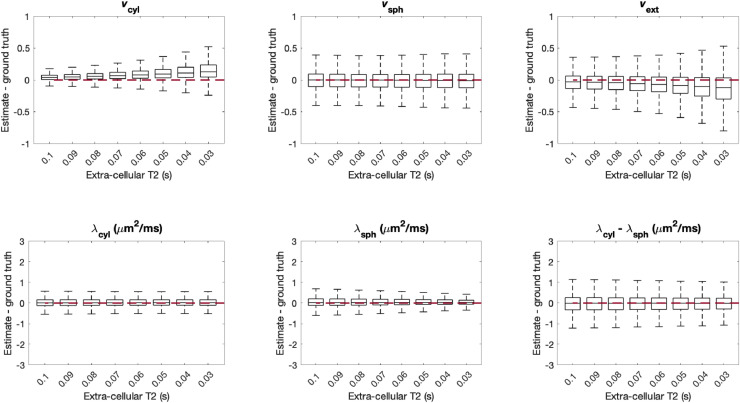
Estimation errors of all parameters when intra-cellular compartments (spherical and cylindrical) and extra-cellular compartments have different T2-relaxation times. For each box, 107,520 sets of parameters were uniformly drawn from the entire plausible parameter space. In these simulations we fixed intra-cellular T2 to 0.1 s and varied extra-cellular T2 between 0.03-0.1 s. As the difference between compartmental T2-values increases, $v_{\text{cyl}}$ tends to be overestimated and $v_{\text{ext}}$ tends to be underestimated. On average, other parameter estimates are largely unaffected by compartmental variation in T2-relaxation.

Fig. 9 shows that as the difference between compartmental T2-values increases, a portion of the extra-cellular signal tends to be attributed to cylindrical compartments. For the largest difference between compartmental T2 values, i.e. when intra-cellular T2 = 0.1 s and extra-cellular T2 = 0.03 s, $v_{\text{cyl}}$ is overestimated by 0.12 and $v_{\text{ext}}$ is underestimated by 0.12 on average. This bias may be particularly relevant in the presence of inflammation, where differences between intra-cellular and extra-cellular T2-relaxation may be exacerbated.

#### 3.2.3 Non-negligible $\lambda_{⊥}^{\text{cyl}}$

Another model assumption we employ is that the perpendicular diffusivity of cylindrical compartments is negligible. To test this, we gradually increased $\lambda_{⊥}^{\text{cyl}},$ noting that Veraart et al. (2020) found that perpendicular diffusivity in cylindrical compartments reflecting biophysically plausible axon sizes is typically less than 0.01 $\mu m^{2}/\text{ms},$ using an 80 mT/m scanner. As before, data was synthesised using 107,520 different parameter combinations sampled uniformly from the entire plausible parameter space.

**Fig. 10:**
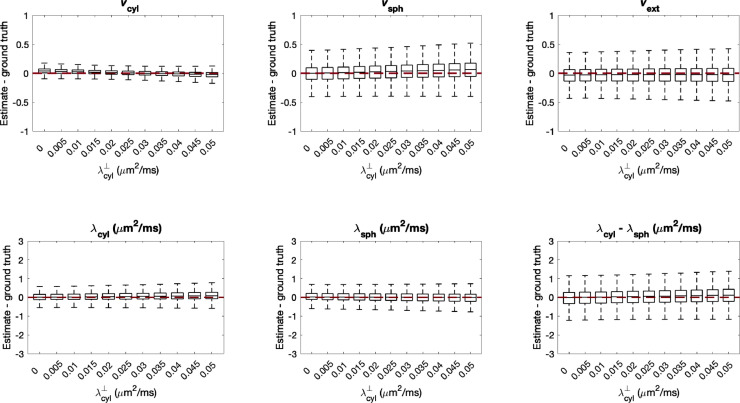
Estimation errors of all parameters when perpendicular diffusivity in cylindrical compartments is non-zero. For each box, 107,520 sets of parameters were uniformly drawn from the entire plausible parameter space. As $\lambda_{⊥}^{\text{cyl}}$ increases, $v_{\text{sph}}$ tends to be overestimated. Veraart et al. (2020) found that perpendicular diffusivity in cylindrical compartments reflecting biophysically plausible axon sizes is typically less than 0.01 $\mu m^{2}/\text{ms},$ using an 80 mT/m scanner corresponding to our experimental set-up.

Fig. 10 shows that for $\lambda_{⊥}^{\text{cyl}}=0.01\;\mu m^{2}/\text{ms},$ the precision of parameter estimates is affected only moderately. In higher gradient strength scanners, or in regions with thicker axons such as in the spinal cord, neglected perpendicular cylindrical diffusivity may lead to higher estimation errors, and in particular may bias $v_{\text{sph}}$.

#### 3.2.4 Myelin compartment

The model does not account for the space occupied by myelin water for which the signal largely decays to zero with the echo times in typical diffusion MRI (MacKay et al., 1994). This may lead to estimation errors, particularly in compartmental volume fractions and in computing extra-cellular diffusivity from the tortuosity approximation. To study the effect of the unaccounted-for myelin volume, we synthesised data sets with an additional myelin compartment which had no associated diffusion signal, satisfying $v_{\text{cyl}}+v_{\text{sph}}+v_{\text{ext}}+v_{\text{myelin}}=1$. We also adjusted the tortuosity model such that $\lambda_{∥}^{\text{ext}}=\lambda v_{\text{ext}}^{\frac{1}{2}v_{\text{sph}}/\left(v_{\text{sph}}+v_{\text{cyl}}+v_{\text{myelin}}\right)}$ and $\lambda_{⊥}^{\text{ext}}=\lambda v_{\text{ext}}^{\left(\frac{1}{2}v_{\text{sph}}+v_{\text{cyl}}+v_{\text{myelin}}\right)/\left(v_{\text{sph}}+v_{\text{cyl}}+v_{\text{myelin}}\right)}$. As in West et al. (2018a), we compare the estimated volume fractions $v_{\text{cyl}},v_{\text{sph}}$ and $v_{\text{ext}}$ to the gold standard values of $v_{\text{cyl}}/\left(1-v_{\text{myelin}}\right),v_{\text{sph}}/\left(1-v_{\text{myelin}}\right)$ and $v_{\text{ext}}/\left(1-v_{\text{myelin}}\right),$ respectively. As before, data was synthesised using 107,520 different parameter combinations sampled uniformly from the entire plausible parameter space.

**Fig. 11:**
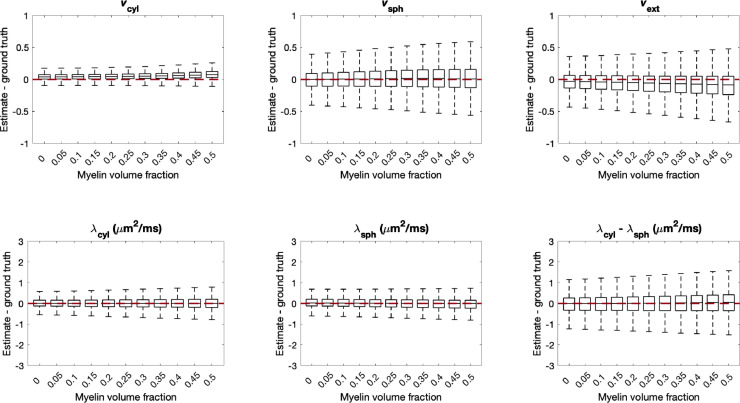
Estimation errors of all parameters when the physical volume of the MR-invisible myelin water pool is considered by incorporating a fourth compartment with no associated MR signal into the test data. For each box, 107,520 sets of parameters were uniformly drawn from the entire plausible parameter space. As myelin volume fraction increases, $v_{\text{cyl}}$ tends to be overestimated and $v_{\text{ext}}$ tends to be underestimated. For grey matter regions where the myelin volume fraction is typically around 0.1 (Laule et al., 2006), the resulting bias is moderate.

Box plots in Fig. 11 show that as the volume fraction of myelin increases in the test data, a portion of the extra-cellular space signal is attributed to cylindrical compartments. In addition, the precision of $v_{\text{cyl}}$ estimates is also reduced. Grey matter typically contains myelin volume fraction of approximately 0.1 (Laule et al., 2006), for which $v_{\text{cyl}}$ is overestimated by 0.041 and $v_{\text{ext}}$ is underestimated by 0.041 on average. In white matter, which features myelin volume fractions of approximately 0.3 (Laule et al., 2006), $v_{\text{cyl}}$ is overestimated by 0.050 and $v_{\text{ext}}$ is underestimated by 0.063 on average.

#### 3.2.5 Tortuosity approximation

Next, we consider the effect of imperfections in modelling extra-cellular space diffusivity. The tortuosity model builds on the idea that parallel to cylindrical compartments, diffusion is only hindered by spherical compartments, while perpendicular to cylindrical compartments, both spherical and cylindrical geometries hinder diffusion. Thus, deviations from the tortuosity approximation in the case of cylindrical compartments would primarily alter $\lambda_{⊥}^{\text{ext}},$ whereas deviations from the tortuosity approximation in the case of spherical compartments would alter both $\lambda_{⊥}^{\text{ext}}$ and $\lambda_{∥}^{\text{ext}}$.

In this section, we investigate the effect of these two deviations from the tortuosity approximation. Firstly, perpendicular extra-cellular diffusivity was perturbed by a factor α, such that $\lambda_{⊥}^{{}^{\prime }\text{ext}}=\min\left(\alpha \lambda_{⊥}^{\text{ext}},\lambda_{\text{free}}\right),$ and the corresponding $\lambda_{∥}^{{}^{\prime }\text{ext}}=\max\left(\lambda_{⊥}^{{}^{\prime }\text{ext}},\lambda_{∥}^{\text{ext}}\right)$. Secondly, both parallel and perpendicular extra-cellular diffusivities were perturbed by a factor β such that $\lambda_{∥}^{{}^{\prime }\text{ext}}=\min\left(\beta \lambda_{∥}^{\text{ext}},\lambda_{\text{free}}\right)$ and $\lambda_{⊥}^{{}^{\prime }\text{ext}}=\min\left(\beta \lambda_{⊥}^{\text{ext}},\lambda_{\text{free}}\right)$. We vary both α and β between [0.5, 1.5], such that when α,β<1, extra-cellular diffusivity is smaller than predicted by the tortuosity approximation, and when α,β>1, extra-cellular diffusivity is larger than predicted by the tortuosity approximation, up to the maximum free diffusivity $\lambda_{\text{free}}$ used in this model. As in the previous sections, data was synthesised using 107,520 different parameter combinations sampled uniformly from the entire plausible parameter space.

**Fig. 12:**
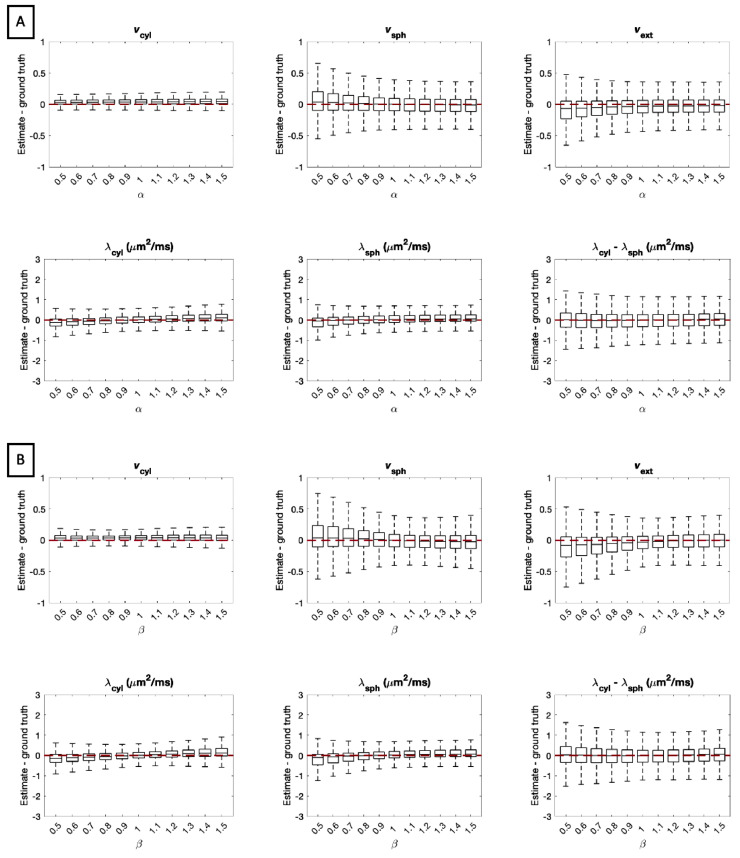
Estimation errors of all parameters when (A) $\lambda_{⊥}^{\text{ext}}$ is perturbed by a factor α and (B) when both $\lambda_{∥}^{\text{ext}}$ and $\lambda_{⊥}^{\text{ext}}$ are perturbed from the tortuosity approximation by a factor β. For each box, 107,520 sets of parameters were uniformly drawn from the entire plausible parameter space. Errors are highest when the mean extra-cellular diffusivity is smaller than predicted by the tortuosity model. In particular, $v_{\text{sph}}$ tends to be overestimated and $v_{\text{ext}}$ tends to be underestimated when α,β<1.

Fig. 12 A and 12 B show the errors when parallel and perpendicular extra-cellular diffusivity is perturbed, respectively. In both cases, errors in apparent volume fraction estimates are minor when extra-cellular diffusivities are higher than predicted by the tortuosity approximation. Meanwhile, when extra-cellular diffusivities are lower than predicted by the tortuosity approximation, the precision of parameter estimates is reduced, and a portion of the extra-cellular signal is attributed to spherical compartments. For all parameters, estimation errors are greater when both $\lambda_{⊥}^{\text{ext}}$ and $\lambda_{∥}^{\text{ext}}$ are misestimated by the tortuosity approximation.

#### 3.2.6 Kurtosis effects

**Fig. 13:**
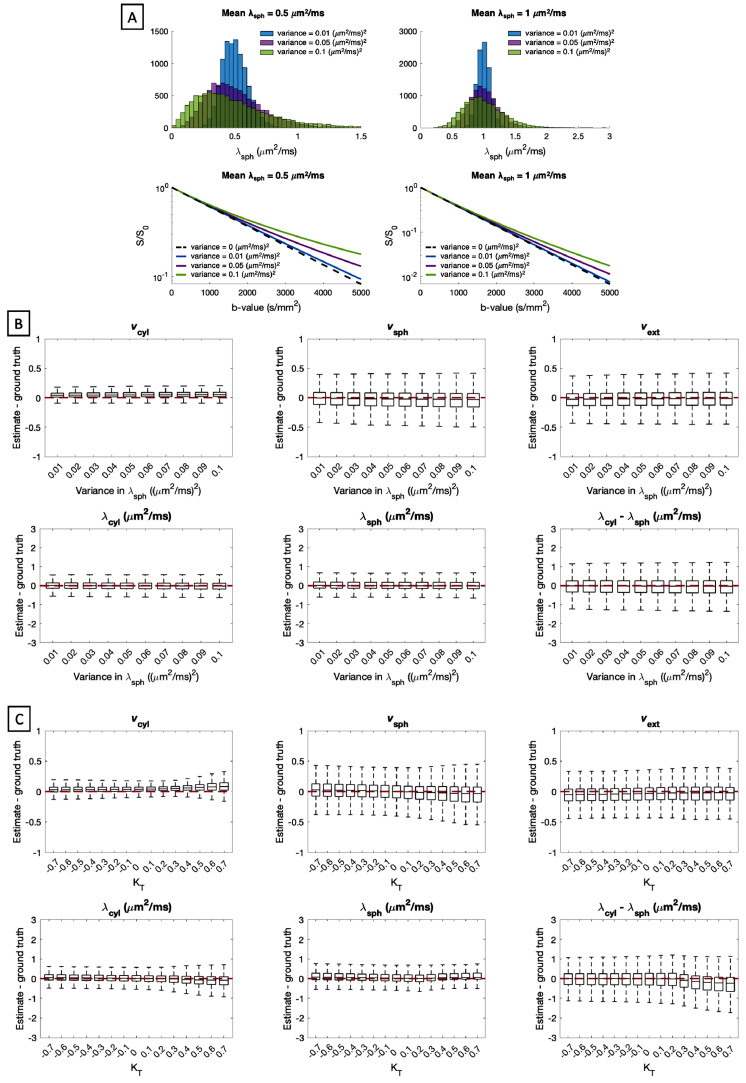
Panel (A): Examples of signal decay curves for different distributions of spherical compartment diffusivities for mean diffusivity of 0.5 $\mu m^{2}/\text{ms}$ (left) and 1 $\mu m^{2}/\text{ms}$ (right). Panel (B): Estimation errors of all parameters when spherical compartment diffusivities are non-uniform and drawn from a gamma distribution with increasing variance. Panel (C): Estimation errors when spherical compartment diffusivities are perturbed by a total kurtosis term $K_{T}$. For each box, 107,520 sets of parameters were uniformly drawn from the entire plausible parameter space.

In this section we probe the scenario where the spherical compartment signal decay is not mono-exponential as assumed by the model, but is confounded by kurtosis. Firstly, we consider the case where spherical compartment diffusivities are not uniform as assumed by the model. To investigate this, we simulated 107,520 samples of the model parameters, $v_{\text{cyl}},v_{\text{sph}},\lambda_{\text{cyl}}$ and $\lambda_{\text{sph}},$ as uniformly as possible. Then, for each $\lambda_{\text{sph}},$ we drew N = 10,000 samples of spherical compartment diffusivities from a gamma distribution with mean set to $\lambda_{\text{sph}},$ and variance between [0, 0.1] ${\left(\mu m^{2}/\text{ms}\right)}^{2}$. The total contribution of spherical compartments to the powder-averaged diffusion signal was the mean signal given by $\frac{v_{\text{sph}}}{N}\sum_{i=1}^{N}\exp\left(-b\lambda_{\text{sph},i}\right)$. We note that in spherical compartments, $b_{\Delta }$ does not affect the signal (see Eq. (9)). In Fig. 13A we show examples of different gamma-distributions of compartment diffusivities for mean diffusivity of 0.5 $\mu m^{2}/\text{ms}$ and 1 $\mu m^{2}/\text{ms},$ as well as the resulting signal decay curves.

Secondly we consider kurtosis in spherical compartment diffusivities more generally. The model used in this work assumes that the diffusion signal arising from spherical compartments is $\exp(-b\lambda_{\text{sph}})$ using both LTE and STE acquisitions. Here, we add a kurtosis term explicitly, such that the spherical compartment diffusion signal takes the form $\exp(-b\lambda_{\text{sph}}+\frac{1}{6}K_{T}b^{2}\lambda_{\text{sph}}^{2})$ where $K_{T}$ refers to total kurtosis, which may arise from several different effects, such as a combination of kurtosis due to restriction and kurtosis due to variance in $\lambda_{\text{sph}}$. To keep the diffusion signal within physically plausible bounds, we constrained $\lambda_{\text{sph}}\leq \frac{3}{b_{\max}K_{T}}$ with $b_{\max}=5000\;s/{\text{mm}}^{2}$. This ensured that the diffusion signal from spherical compartments was monotonically decreasing up to the maximum b-value of 5000 $s/{\text{mm}}^{2}$ used in this work. Using this we simulated 107,520 samples, keeping the other model parameters as uniform as possible. Jespersen et al. (2019) and Henriques et al. (2020) suggested that in highly restricted compartments, kurtosis due to restriction may be negative, whereas kurtosis due to variance in $\lambda_{\text{sph}}$ is positive as shown in Fig. 13A. Henriques et al. (2020) reported total kurtosis of approximately 0.7 in in-vivo rat brain. Here, we extend the analysis to negative kurtosis values as well to account for the potential scenario where there is negligible kurtosis due to variance in $\lambda_{\text{sph}}$ and substantial negative kurtosis due to restriction. Thus, we varied $K_{T}$ between [-0.7, 0.7].

Results for the two kurtosis effects are shown in Fig. 13B and Fig. 13C. Fig. 13B shows that as the variance in $\lambda_{\text{sph}}$ increases, a portion of the signal from spherical compartments is attributed to cylindrical compartments and extra-cellular space, and that the precision of compartment volume fraction estimates is adversely affected by wide distributions of $\lambda_{\text{sph}}$. Fig. 13C shows that when $K_{T}<0$ a portion of the signal from extra-cellular space is attributed to spherical compartments, and $\lambda_{\text{cyl}}$ and $\lambda_{\text{sph}}$ are slightly overestimated on average. When $K_{T}>0,$ a portion of the signal from spherical compartments is attributed to cylindrical compartments, $\lambda_{\text{sph}}$ is overestimated and $\lambda_{\text{cyl}}$ is underestimated on average.

#### 3.2.7 Summary of key effects

The list below summarises the key effects that various departures from the idealised model have on parameter estimates.

• At the interface between CSF and white matter, a large portion of the CSF signal is attributed to spherical compartments.
• The assumption that T2-relaxation is the same in extra- and intra-cellular compartments could lead to a portion of the extra-cellular signal being attributed to cylindrical compartments, which could be an important issue in the presence of inflammation.
• When $\lambda_{⊥}^{\text{cyl}}$ is non-negligible, a portion of the cylindrical compartment signal may be attributed to spherical compartments. Higher errors may be expected in the presence of thicker axons such as in the spinal cord, or when ultra-high gradient strengths are used for measurement.
• In the presence of myelin, a small portion of the extra-cellular space signal may be attributed to cylindrical compartments.
• When the diffusivity of extra-cellular space is smaller than predicted by the tortuosity approximation, a portion of the extra-cellular space signal may be attributed to spherical compartments.
• When there is positive kurtosis in spherical compartment diffusivities, a portion of the signal from spherical compartments is attributed to cylindrical compartments, whereas when there is negative kurtosis in spherical compartment diffusivities, a portion of the signal from extra-cellular compartments is attributed to spherical compartments.

### 3.3 In-vivo data characterisation

#### 3.3.1 Model fitting

**Fig. 14:**
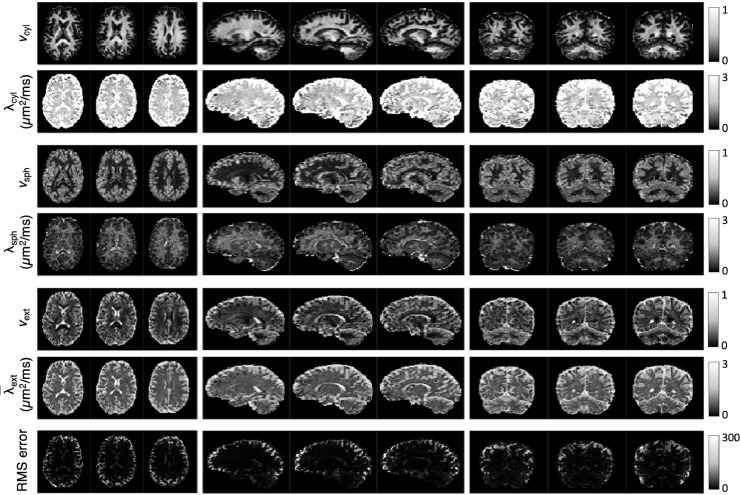
Apparent volume fraction and diffusivity of microscopic compartments in brain grey and white matter for subject 1. The estimated microstructural parameters are unconfounded by orientation heterogeneity in tissue. The contrast in these maps indicates that overall the apparent volume fraction of cylindrical compartments ($v_{\text{cyl}}$) is higher in white matter than in grey matter and the apparent volume fraction of spherical compartments ($v_{\text{sph}}$) is higher in grey matter than in white matter. In the last row we show the root-mean-square estimation error with respect to the powder-averaged diffusion measurements.

After testing the accuracy of parameter estimation with simulations, we now estimate the apparent volume fractions of cylindrical and spherical compartments ($v_{\text{cyl}}$ and $v_{\text{sph}},$ respectively), and the apparent diffusivities of cylindrical and spherical compartments ($\lambda_{\text{cyl}}$ and $\lambda_{\text{sph}},$ respectively) in-vivo. Resulting maps for one of the subjects are shown in Fig. 14, and maps for the other subjects can be found in the Supplementary Material (Figs. S5–S9). The SNR in these in-vivo maps is approximately 25. Contrast in the parameter maps show that, in general, $v_{\text{cyl}}$ is higher in white matter than in grey matter and $v_{\text{sph}}$ is higher in grey matter than in white matter. $v_{\text{sph}}$ and $v_{\text{cyl}}$ are close to zero in the ventricles, suggesting that CSF is encapsulated in the extra-cellular compartment, which in the absence of cell structures behaves like a region of free water.

To investigate the microscopic parameter estimates further, the brain of each subject was segmented. Eight regions of interest were selected from the segmentation, namely the left and right halves of cerebral white and grey matter, as well as the left and right halves of cerebellar white and grey matter. The outermost voxels of each region-of-interest mask were removed to eliminate noticeable partial volume effects and imperfections in the coregistration between the diffusion data and the T1-weighted image.

**Fig. 15:**
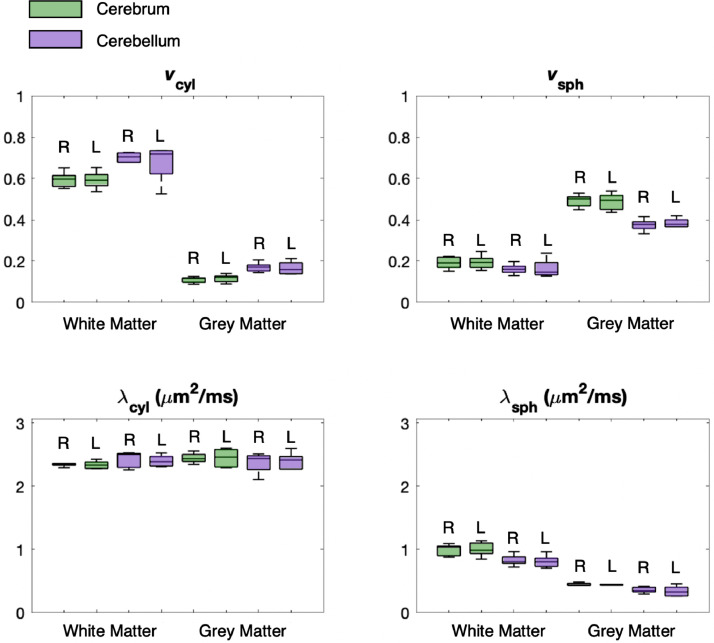
Regional analysis of spherical and cylindrical apparent compartment volume fractions and diffusivities for all subjects. Green represents the cerebrum and purple represents the cerebellum, while R and L mark the right and left sides of the brain, respectively. On average, the apparent volume fraction of cylindrical compartments ($v_{\text{cyl}}$) is higher in white matter and the apparent volume fraction of spherical compartments ($v_{\text{sph}}$) is higher in grey matter. Due to low compartmental volume fractions, estimates of $\lambda_{\text{sph}}$ may be unreliable in white matter as shown in Figs. 4 and 5. (For interpretation of the references to colour in this figure legend, the reader is referred to the web version of this article.)

Fig. 15 shows box plots of the mean parameter values estimated for each subject for the different regions. For estimates of $\lambda_{\text{cyl}},$ differences between the segmented brain regions are not statistically significant, whereas for the other three parameters, differences between grey matter and white matter are high with p-values $<10^{-10}$. Differences between estimates in the cerebrum and cerebellum are also statistically significant for all parameter values except for $\lambda_{\text{cyl}}$. $v_{\text{cyl}}$ is higher in the cerebellum than in the cerebrum both in white matter and grey matter. In grey matter this may indicate the presence of large dendritic arbours associated with Purkinje cells in the cerebellum, whereas in white matter this difference may be due to fine mossy and climbing fibres (Johns, 2014, Shepherd, 1998). In grey matter, $v_{\text{sph}}$ is lower in the cerebellum than in the cerebrum. The cerebellar cortex contains a densely packed layer of granule cells, which is not reflected by our results likely because the voxels in the present study are too large to resolve this layer. The signature of granule cells, which are typically small (≲10μm in diameter) compared to soma sizes in the cerebral cortex (∼8−16μm in diameter) (Beebe, Young, Mellott, Schofield, 2016, von Economo, 2009, Johns, 2014, Shepherd, 1998), may be captured by spherical compartment diffusivity, which is lower in the cerebellar cortex than in the cerebral cortex, and which has also been detected by Tax et al. (2020). $\lambda_{\text{sph}}$ also appears to be higher in white matter than in grey matter. However, white matter features low $v_{\text{sph}},$ which makes the estimation of $\lambda_{\text{sph}}$ inherently unreliable in white matter, as demonstrated in Figs. 4 and 5. Finally, differences between the left and right brain regions are not statistically significant for any of the parameter estimates. In Fig. S10 we visualise the density of parameter combinations estimated in-vivo throughout the parameter space in a similar format to Figs. 3 and 4 for comparison.

#### 3.3.2 Recovering fibre orientation

**Fig. 16:**
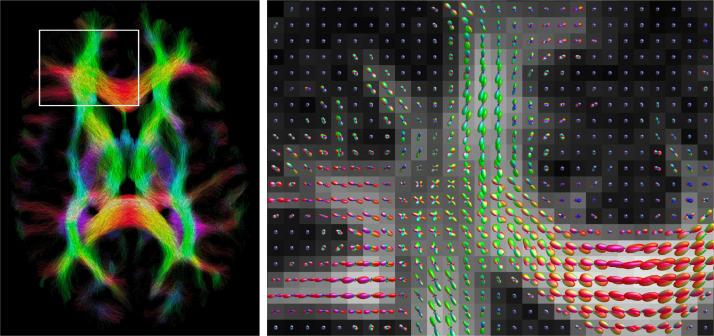
Fibre tracts for a whole transverse slice (left) and fibre orientation distribution zoomed in onto a region of crossing fibres near the genu of the corpus callosum. The fibre orientation distribution was estimated using spherical deconvolution with a spatially varying response function determined from the recovered microstructural parameters. Connectivity was mapped using probabilistic tractography (Tournier et al., 2010). The underlying map represents the conventional fractional anisotropy recovered from DTI. The figure demonstrates that the technique presented in this work can also be used to recover fibre orientation distribution and to map the brain’s circuitry. (For interpretation of the references to colour in this figure legend, the reader is referred to the web version of this article.)

By recovering the apparent volume fraction and diffusivity of neural projections, it is possible to determine the fibre orientation distribution P(ϕ) using Eq. (7) and the orientation-sensitive LTE data. We demonstrate this in Fig. 16, where we show the fibre orientation distribution and tractography results that reflect connectivity in a healthy subject, visualised with MRtrix3 (Tournier et al., 2019). We show a zoomed region where crossing fibres are correctly identified near the genu of the corpus callosum. To recover the fibre orientation distribution we performed spherical deconvolution with a spatially varying response function and spherical harmonics basis with maximum order of 8 (Kaden, Kelm, Carson, Does, Alexander, 2016b, Kaden, Kruggel, Alexander, 2016a). The estimated fibre orientation distribution was constrained to be non-negative and normalised to unity. After recovering the fibre orientation distribution, we performed probabilistic tractography (Tournier et al., 2010) to map connectivity in the brain.

### 3.4 Clinically viable acquisition time

**Fig. 17:**
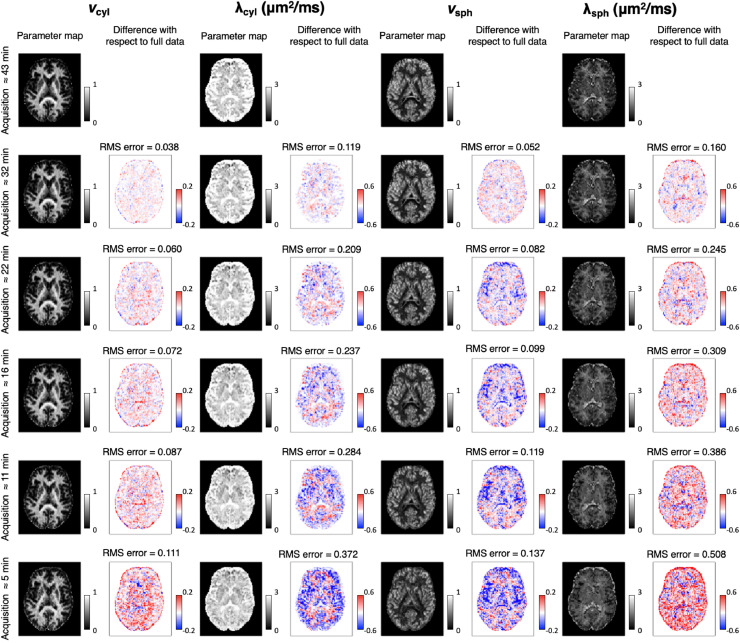
To achieve practical acquisition times, we reduce the full 43 min data set. This figure shows the difference between parameter estimation using the full data set and data where the number of STE and LTE measurements are equally reduced. The maps of the parameter estimation are shown in grey scale, and the difference from the full data with a red-blue scale. The RMS error is shown at the top of each error plot. The full data set corresponds to 128 gradient directions for both STE and LTE, and the reduced data corresponds to 96, 64, 48, 32 and 16 gradient directions in total for STE and LTE each. The plots are shown for subject 1. (For interpretation of the references to colour in this figure legend, the reader is referred to the web version of this article.)

The methods presented in this work were designed for a widely available 3T MRI scanner. The b-values we used are achievable with TE = 94 ms, which also yields a reasonable SNR of 25. For a full brain diffusion-weighted scan with 2 mm isotropic resolution, the acquisition time is 43 min. To make the developed methods achievable with more practical scan times on a 3T scanner, the data used for parameter estimation was reduced by removing gradient directions across all b-shells equally between STE and LTE. From the full data set of 128 gradient directions for both STE and LTE, data sets with 96, 64, 48, 32 and 16 uniformly distributed gradient directions were created. Maps recovered from the reduced data are compared to the maps from the full data in Fig. 17. This figure demonstrates that parameter estimates degrade gracefully as the data set reduces in size and contrast remains largely consistent with the full data set, which raises the prospect of more practical acquisition times. However, it should be noted that the errors increase systematically in the maps from reduced data sets. Lower parameter values tend to be overestimated, and higher parameter values tend to be underestimated, which overall reduces contrast in the parameter maps.

## 4 Discussion

### 4.1 Contributions

This work explores the potential to disentangle quasi-spherical and quasi-cylindrical cellular structures in the human brain using a clinically viable 3T MRI protocol with feasible b-values, SNR and scan times. Using a simple biophysical model that considers the diffusion signature of spherical and cylindrical compartments, we show in-vivo parameter maps that reflect the presence of these geometries in brain tissue. Our maps show that the volume fraction of spherical compartments ($v_{\text{sph}}$) is higher in grey matter than white matter, which is in good agreement with recent works using a Connectome scanner with ultra-high gradient strength (Palombo, Ianus, Guerreri, Nunes, Alexander, Shemesh, Zhang, 2020, Tax, Szczepankiewicz, Nilsson, Jones, 2020), reinforcing the suggestion that $v_{\text{sph}}$ may provide a marker for the density of neuronal and glial cell bodies. Crucially, however, our approach does not require ultra-high gradient strength and is achievable on widely available MRI scanners. Our maps also show a low, but non-negligible, cylindrical compartment density ($v_{\text{cyl}}$) in grey matter. This is consistent with previous findings and suggestions that the diffusion signal may not fully capture dendrites but is more sensitive to axons (Lampinen et al., 2019), and highlights the need for future investigation. For spherical compartments, $\lambda_{\text{sph}}$ provides an apparent diffusivity that is likely influenced by several tissue properties, such as bulk diffusivity, cell size distribution and exchange processes between quasi-spherical and quasi-cylindrical compartments, for example water exchanging between the cell soma and its projections. Limited by data availability in practical in-vivo settings, it is challenging to disentangle these different effects, and hence we do not ascribe a specific biological meaning to $\lambda_{\text{sph}}$. Fig. 4 shows that estimates of $\lambda_{\text{sph}}$ may be unreliable in white matter due to low $v_{\text{sph}}$ in white matter. Simulations show that the biophysical model provides parameter estimates that are reasonably robust to departures from the modelling assumptions, particularly in the absence of substantial partial volume effects with CSF. We highlight that for estimating the four independent parameters of the model used in this work, higher b-values such as up to 5000 $s/{\text{mm}}^{2}$ with LTE are beneficial and easily achievable within an echo time of 94 ms. The developed technique also provides useful contrast in white matter, where our maps show high cylindrical compartment density, likely reflecting neural projection density consistent with existing techniques (Kaden, Kelm, Carson, Does, Alexander, 2016b, Lampinen, Szczepankiewicz, Martensson, van Westen, Sundgren, Nilsson, 2017, Zhang, Schneider, Wheeler-Kingshott, Alexander, 2012), and a low, but non-negligible, spherical compartment density. Furthermore, the technique can be used to recover fibre orientation distribution, potentially supporting combined tractography and parameter mapping to enhance future connectivity-mapping and ”tractometry” in the brain (Chamberland et al., 2019).

Two key technical advances combine to enable the maps we describe above: B-tensor encoding measurements and machine-learning based parameter mapping. Firstly, to facilitate estimation of apparent markers of distinct cellular structures in potential in-vivo settings on a 3T scanner, we use richer diffusion data in the form of B-tensor encoding. Specifically, we acquired data with spherical tensor encoding and linear tensor encoding gradient waveforms. This was motivated by the contrast inversion effect shown in Fig. 1, which suggests particular sensitivity to grey matter morphology. Although the measurement protocol we use here requires 43 min, Section 3.4 shows little penalty with, for example, a 32 min protocol, which could be reduced to approximately 10 min using multiband EPI with an acceleration factor of 3, noting that simultaneous multislice methods with moderate acceleration factors do not lead to significant reduction in SNR (Auerbach, Xu, Yacoub, Moeller, Ugurbil, 2013, Setsompop, Gagoski, Polimeni, Witzel, Wedeen, Wald, 2012). Secondly, to facilitate the fast and robust estimation of model parameters, we developed an artificial neural network which we trained on synthetic data generated with the biophysical model. We injected noise with variable σ to the training data, and hence the estimation procedure has an intrinsic denoising effect on the parameter maps. The most time-consuming element of this approach is the network training, which only needs to be done once for an experimental protocol determined by the number and type of B-tensor measurements. Parameter mapping at test time then requires only seconds for full 3D images. Other experimental parameters including noise, spatial resolution and gradient directions can be freely varied without needing to retrain the neural network.

### 4.2 Limitations

In this work, we adopt a simple biophysical model. While this model does not accommodate many subtle effects that affect the MR signal from neural tissue, the aim is to capture the key influences from cellular structures to obtain approximate maps of their density. We emphasise that the model does not fully quantify such densities but is designed instead to reflect contrast in these kinds of geometric entities. The simplicity of the model is essential to ensure stable parameter estimates particularly given clinically viable data. Violations of the assumptions of such models always arise in practice, but we believe the simpler the model, the easier it is to interpret parameter values even in the presence of assumption violation. To aid that interpretation we run a variety of simulation experiments to highlight the effects of violations likely to arise in practice. The next few paragraphs summarise the key effects of model departures on the obtained maps.

The model assumes that T2-relaxation does not significantly differ among tissue compartments within a voxel, noting that the signal from the myelin water pool is largely decayed at the relatively long echo time of a typical diffusion MRI scan. Clark and Le Bihan (2000) showed that this is indeed a reasonable approximation in the healthy human brain, but other studies such as McKinnon and Jensen (2019); Veraart et al. (2018) found differences between intra- and extra-compartmental T2-relaxation times. We used simulations to test this further, which demonstrate that differences in T2-relaxation between intra-cellular (spherical and cylindrical structures) and extra-cellular compartments affect $v_{\text{sph}}$ and $\lambda_{\text{sph}}$ very little, but manifests as a bias in $v_{\text{cyl}}$ as differences become large. However, in the case of certain neuropathological conditions, it might be advisable to acquire additional data to assess potential differences in T2-relaxation between cellular compartments (Lampinen et al., 2019).

In cortical grey matter, partial volume effects with CSF may also bias parameter estimates due to longer T2-relaxation in CSF relative to tissue. We quantify this effect with simulations and show that in the presence of CSF, a large portion of the CSF signal may be attributed to spherical compartments. Our results highlight that estimation errors are stronger in regions with high $v_{\text{cyl}}$ compared to regions with low $v_{\text{cyl}},$ suggesting that CSF contamination effects are less prevalent at the boundary between CSF and grey matter, such as near the subarachnoid space, compared to the boundary between CSF and white matter, such as near the ventricles. Furthermore, Supplementary Figure S11 shows preliminary results using fluid-attenuated inversion recovery (FLAIR) to suppress the CSF signal. The figure compares parameter estimates acquired with and without CSF suppression and demonstrates that while CSF suppression has an effect on parameter estimates, the overall contrast observed in $v_{\text{sph}}$ maps remains consistent with those without CSF suppression, suggesting that it does not arise purely from partial volume.

The biophysical model assumes that $\lambda_{⊥}^{\text{cyl}}$ is negligible. Indeed, Veraart et al. (2020) found that, using gradient strengths up to 80 mT/m as in the present work, typical values of $\lambda_{⊥}^{\text{cyl}}$ are less than 0.01 $\mu m^{2}/\text{ms}$. For such small diffusivities, we observe only small biases in our parameter estimates as revealed in Section 3.2.3. However, if higher gradient strengths are used or tissues with thicker cylindrical compartments are scanned, such as axons in the spinal cord, the assumption that $\lambda_{⊥}^{\text{cyl}}=0$ may lead to a portion of the signal from cylindrical compartments to be attributed to spherical compartments.

In this work, we do not consider myelin content in the brain, as we expect the signal from myelin water to be negligible at the echo time used in this work. However, the physical space occupied by myelin may still impact our parameter estimates, for example via the tortuosity approximation, which is dependent on the geometrical configuration of differently shaped cellular structures. We investigated how a myelin compartment may impact our parameter estimates in Section 3.2.4 and found that $v_{\text{cyl}}$ may be over-estimated in the presence of substantial myelin fraction. We did not observe large biases in $v_{\text{sph}}$ or the compartment diffusivities, but show increased variance in these parameter estimates as myelin content increases. These estimation errors are likely to be greater in white matter, where myelin content is expected to be high, compared to grey matter.

The tortuosity approximation used to characterise diffusion in extra-cellular space assumes that a microenvironment explored by diffusing water may only contain spherical structures and collinear cylindrical structures. This may not be true in extra-cellular space near branching dendritic trees in grey matter, where cylindrical structures may not be parallel to one another even within the small region explored by water molecules. The tortuosity model is a first-order approximation, which is relatively accurate for low volume fractions of spherical and cylindrical structures, but may show larger deviations in the case of higher volume fractions (Novikov et al., 2018), depending on tissue properties such as size distribution and packing geometry. In light of this, we investigated deviations from the tortuosity approximation by perturbing both the parallel and perpendicular extra-cellular diffusivities and found that spherical compartment content may be over-estimated when the extra-cellular diffusion tensor is substantially smaller than predicted by the tortuosity approximation. Conversely, when extra-cellular diffusivity was higher than predicted by the tortuosity approximation, the bias in parameter estimates is moderate.

Kurtosis in the T2-normalised diffusion signal decay may arise from several different tissue properties. Henriques et al. (2020) identify three potential sources: (i) compartment shape anisotropy, (ii) variance in compartment mean diffusivities and (iii) geometrical restriction, and we elaborate on how each of these may affect our results. Regarding (i), the biophysical model used in this work explicitly accounts for kurtosis arising from compartment shape anisotropy. Eq. (9) shows that in measurements where $b_{\Delta }\neq 0,$ such as LTE, the signal powder average contains an error function term that manifests as kurtosis provided that the parallel and perpendicular diffusivities of a compartment are not equal. Regarding kurtosis source (ii), the model takes into account combinations of spherical, cylindrical and extra-cellular compartments, which are expected to have different mean diffusivities, and hence give rise to kurtosis in the signal decay. However, the model does not consider variance of mean diffusivities of individual compartments, for example in the presence of different cell sizes. To study how this model limitation affects our parameter estimates, we simulated gamma-distributed spherical compartment diffusivities in Section 3.2.6 and show that $v_{\text{sph}}$ may be underestimated in the presence of wide soma size distributions. Finally, to probe the effect of source (iii), we explicitly added a kurtosis term to the diffusion signature of spherical compartments. When kurtosis is negative, such as potentially in the presence of highly restricted compartments (Henriques, Jespersen, Shemesh, 2020, Jespersen, Olesen, Ianus, Shemesh, 2019), $v_{\text{sph}}$ tends to be slightly overestimated, whereas when kurtosis is positive, $v_{\text{sph}}$ tends to be slightly underestimated.

The diffusion encoding STE and LTE waveforms used in this work have different spectral content, which could lead to unwanted time-dependence effects (Grebenkov, 2010, Jespersen, Olesen, Ianus, Shemesh, 2019). Indeed, the Monte-Carlo simulations we show in the Supplementary Material (Fig. S12B) demonstrate that in impermeable spherical compartments, STE and LTE signals have substantially different apparent diffusivity, consistent with findings in Lundell et al. (2019). However, we do not observe the same effect in in-vivo measurements, as shown by signal decay curves in Fig. S12A. Based on simulation results with quasi-spherical meshes containing quasi-cylindrical protrusions in Fig. S12C, we suggest that the discrepancy between in-vivo measurements and a restriction model may be due to exchange processes between cell bodies and projections. This result motivates the use of an apparent spherical compartment diffusivity which encapsulates multiple properties of cell bodies, including cell size, bulk diffusivity and water exchange between cellular compartments, instead of a restriction model. The effect of water exchange between cellular compartments could be explicitly incorporated in the biophysical model similar to Stanisz et al. (1997). However, this would require estimating additional independent parameters, which is challenging with a clinically viable MRI protocol due to limitations in acquisition time and data sensitivity.

### 4.3 Future work

Rigorous validation and evaluation against histology both in health and disease remain important for drawing robust conclusions from contrasts observed in the maps the technique provides. We note, however, that Stanisz et al. (1997) provide histological validation for a similar 3-compartment model used to characterise axons and glial cell bodies in bovine optic nerve. More recently, Palombo et al. (2019) suggested good agreement between soma fractions obtained in histology and using the SANDI model in mouse brain. However, the isotropic compartment used to detect soma in this work may also capture other quasi-spherical entities in tissue, such as vacuoles, prevalent for example in prion diseases (Figini et al., 2015), and synaptic boutons, which are typically small (Innocenti and Caminiti, 2016). Measuring the contribution of these structures to the diffusion signal remains future work.

Future work might also tailor the technique to specific neuropathological conditions. In conditions where cortical microstructure may be disrupted, such as in focal cortical dysplasia (Adler et al., 2017), it may be desirable to explicitly model CSF, or alternatively to suppress its signal contribution using FLAIR as shown in preliminary results in the Supplementary Material, Fig. S11. Meanwhile, conditions that feature tissue inflammation, such as multiple sclerosis (Bagnato et al., 2019), may require estimation of compartment-specific T1- and T2-relaxation times. This could be achieved by obtaining measurements with varying echo times and inversion times.

To reduce partial volume effects between cortical grey matter and the surrounding CSF and white matter, increasing the resolution of the diffusion images would be beneficial. To achieve this within practical acquisition times, data could be acquired using multiband EPI acceleration (Auerbach, Xu, Yacoub, Moeller, Ugurbil, 2013, Setsompop, Gagoski, Polimeni, Witzel, Wedeen, Wald, 2012). Additionally, different B-tensor encoding combinations could be used, such as LTE and PTE, which facilitate high b-value measurements at lower echo times, as shown in Fig. S11. Reisert et al. (2019) showed that to resolve degeneracy in certain conditions, a combination of LTE and PTE may be advantageous over a combination of LTE and STE. Future work will explore the impact of these ideas.

## CRediT authorship contribution statement

**Noemi G. Gyori:** Methodology, Formal analysis, Investigation, Writing - original draft, Visualization. **Christopher A. Clark:** Resources, Writing - review & editing, Supervision, Funding acquisition. **Daniel C. Alexander:** Resources, Writing - review & editing, Supervision, Funding acquisition. **Enrico Kaden:** Conceptualization, Methodology, Software, Writing - review & editing, Supervision, Funding acquisition.

## Appendix A Supplementary materials

Supplementary Data S1 Supplementary Raw Research Data. This is open data under the CC BY license http://creativecommons.org/licenses/by/4.0/ Supplementary Data S1

## References

[bib0001] Acosta-Cabronero J., Nestor P.J. (2014). Diffusion tensor imaging in Alzheimer’s disease: insights into the limbic-diencephalic network and methodological considerations. Front. Aging Neurosci..

[bib0002] Adler S., Lorio S., Jacques T.S., Benova B., Gunny R., Cross J.H., Baldeweg T., Carmichael D.W. (2017). Towards in vivo focal cortical dysplasia phenotyping using quantitative MRI. Neuroimage Clin..

[bib0003] Alexander D.C., Dyrby T.B., Nilsson M., Zhang H. (2019). Imaging brain microstructure with diffusion MRI: practicality and applications. NMR Biomed..

[bib0004] Ameis S.H., Lerch J.P., Taylor M.J., Lee W., Viviano J.D., Pipitone J., Nazeri A., Croarkin P.E., Voineskos A.N., Lai M., Crosbie J., Brian J., Soreni N., Schachar R., Szatmari P., Arnold P.D., Anagnostou E. (2016). A diffusion tensor imaging study in children with ADHD, autism spectrum disorder, OCD, and matched controls: distinct and non-distinct white matter disruption and dimensional brain-behaviour relationships. Am. J. Psychiatry.

[bib0005] Andersson J.L.R., Skare S., Ashburner J. (2003). How to correct susceptibility distortions in spin-echo echo-planar images: application to diffusion tensor imaging. Neuroimage.

[bib0006] Andersson J.L.R., Sotiropoulos S.N. (2016). An integrated approach to correction for off-resonance effects and subject movement in diffusion MR imaging. Neuroimage.

[bib0007] Assaf Y. (2019). Imaging laminar structures in the gray matter with diffusion MRI. Neuroimage.

[bib0008] Assaf Y., Basser P.J. (2005). Composite hindered and restricted model of diffusion (charmed) MR imaging of the human brain. Neuroimage.

[bib0009] Auerbach E.J., Xu J., Yacoub E., Moeller S., Ugurbil K. (2013). Multiband accelerated spin-echo echo planar imaging with reduced peak RF power using time-shifted RF pulses. Magn. Reson. Med..

[bib0010] Bagnato F., Franco G., Li H., Kaden E., Ye F., Fan R., Chen A., Alexander D.C., Smith S.A., Dortch R., Xu J. (2019). Probing axons using multi-compartmental diffusion in multiple sclerosis. Ann. Clin. Transl. Neurol..

[bib0011] Basser P.J., Mattiello J., LeBihan D. (1994). Estimation of the effective self-diffusion *tensor* from the NMR spin echo. J. Magn. Reson..

[bib0012] Beebe N.L., Young J.W., Mellott J.G., Schofield B.R. (2016). Extracellular molecular markers and soma size of inhibitory neurons: evidence for four subtypes of gabaergic cells in the inferior colliculus. J. Neurosci..

[bib0013] Behrens T.E.J., Woolrich M.W., Jenkinson M., Johansen-Berg H., Nunes R.G., Clare S., Matthews P.M., Brady J.M., Smith S.M. (2003). Characterization and propagation of uncertainty in diffusion-weighted mr imaging. Magn. Reson. Med..

[bib0014] Bender B., Klose U. (2009). Cerebrospinal fluid and interstitial fluid volume measurements in the human brain at 3T with EPI. Magn. Reson. Med..

[bib0015] Callaghan P.T., Komlosh M.E. (2002). Locally anisotropic motion in a macroscopically isotropic system: displacement correlations measured using double pulsed gradient spin-echo NMR. Magn. Reson. Chem..

[bib0016] Callaghan P.T., Soderman O. (1983). Examination of the lamellar phase of aerosol ot/water using pulsed field gradient nuclear magnetic resonance. J. Phys. Chem..

[bib0017] Caruyer E., Lenglet C., Sapiro G., Deriche R. (2013). Design of multishell sampling schemes with uniform coverage in diffusion MRI. Magn. Reson. Med..

[bib0018] Chamberland M., Raven E.P., Genc S., Duffy K., Descoteaux M., Parker G.D., Tax C.M.W., Jones D.K. (2019). Dimensionality reduction of diffusion MRI measures for improved tractometry of the human brain. Neuroimage.

[bib0019] Cheng Y., Cory D.G. (1999). Multiple scattering by NMR. J. Am. Chem. Soc..

[bib0020] Ciccarelli O., Werring D.J., Wheeler-Kingshott C.A.M., Barker G.J., Parker G.J.M., Thompson A.J., Miller D.H. (2001). Investigation of ms normal-appearing brain using diffusion tensor MRI with clinical correlations. Neurology.

[bib0021] Clark C.A., Barrick T.R., Murphy M.M., Bell B.A. (2003). White matter fiber tracking in patients with space-occupying lesions of the brain: a new technique for neurosurgical planning?. Neuroimage.

[bib0022] Clark C.A., Le Bihan D. (2000). Water diffusion compartmentation and anisotropy at high B values in the human brain. Magn. Reson. Med..

[bib0023] Clayden J.D. (2013). Imaging connectivity: MRI and the structural networks of the brain. Funct. Neurol..

[bib0024] Coelho S., Pozo J.M., Jespersen S.N., Jones D.K., Frangi A.F. (2019). Resolving degeneracy in diffusion MRI biophysical model parameter estimation using double diffusion encoding. Magn. Reson. Med..

[bib0025] Conturo T.E., Lori N.F., Cull T.S., Akbudak E., Snyder A.Z., Shimony J.S., McKinstry R.C., Burton H., Raichle M.E. (1999). Tracking neuronal fiber pathways in the living human brain. Proc. Natl. Acad. Sci. U.S.A..

[bib0026] Cooper H.E., Kaden E., Halliday L.F., Bamiou D.-E., Mankad K., Peters C., Clark C.A. (2019). White matter microstructural abnormalities in children with severe congenital hypothyroidism. Neuroimage Clin..

[bib0027] Cory D.G., Garroway A.N., Miller J.B. (1990). Applications of spin transport as a probe of a local geometry.

[bib0029] Dempster K., Norman R., Theberge J., Densmore M., Schaefer B., Williamson P. (2017). Cognitive performance is associated with gray matter decline in first episode psychosis. Psychiatry Res. Neuroimaging.

[bib0030] Drobnjak I., Zhang H., Ianus A., Kaden E., Alexander D.C. (2016). PGSE, OGSE, and sensitivity to axon diameter in diffusion MRI: insight from a simulation study. Magn. Reson. Med..

[bib0031] von Economo C. (2009). Cellular Structure of the Human Cerebral Cortex.

[bib0032] Eriksson S., Lasic S., Nilsson M., Westin C.-F., Topgaard D. (2015). NMR diffusion-encoding with axial symmetry and variable anisotropy: distinguishing between prolate and oblate microscopic diffusion tensors with unknown orientation distribution. J. Chem. Phys..

[bib0033] Eriksson S., Lasic S., Topgaard D. (2013). Isotropic diffusion weighting in PGSE NMR by magic-angle spinning of the q-vector. J. Magn. Reson..

[bib0034] Ernst T., Kreis R., Ross B.D. (1993). Absolute quantitation of water and metabolites in the human brain. I. Compartments and water. J. Magn. Reson. Ser. B.

[bib0035] Eshagi A., Prados F., Brownlee W.J., Altmann D.R., Tur C., Cardoso M.J., De Angelis F., van de Pavert S.H., Cawley N., De Stefano N., Stromillo M.L., Battaglini M., Ruggieri S., Gasperini C., Filippi M., Rocca M.A., Rovira A., Sastre-Garriga J., Vrenken H., Leurs C.E., Killestein J., Pipamer L., Enzinger C., Ourselin S., Gandini Wheeler-Kingshott C.A.M., Chard D., Thompson A.J., Alexander D.C., Barkhof F., Ciccarelli O. (2018). Deep gray matter volume loss drives disability worsening in multiple sclerosis. Ann. Neurol..

[bib0036] Essayed W.I., Zhang F., Unadkat P., Rees Cosgrove G., Golby A.J., O’Donnell L.J. (2017). White matter tractography for neurosurgical planning: a topography-based review of the current state of the art. Neuroimage Clin..

[bib0037] Fieremans E., Jensen J.H., Helpern J.A. (2011). White matter characterization with diffusional kurtosis imaging. Neuroimage.

[bib0038] Figini M., Alexander D.C., Redaelli V., Fasano F., Grisoli M., Baselli G., Gambetti O., Tagliavini F., Bizzi A. (2015). Mathematical models for the diffusion magnetic resonance signal abnormality in patients with prion diseases. Neuroimage Clin..

[bib0039] Finsterbusch J., Koch M.A. (2008). A tensor approach to double wave vector diffusion-weighting experiments on restricted diffusion. J. Magn. Reson..

[bib0040] Fischl B. (2012). Freesurfer. Neuroimage.

[bib0041] Fung S.H., Roccatagliata L., Gonzalez R.G., Schaefer P.W. (2011). Mr diffusion imaging in ischemic stroke. Neuroimaging Clin. N. Am..

[bib0042] Gellersen H.M., Guo C.C., O’Callaghan C., Tan R.H., Sami S., Hornberger M. (2017). Cerebellar atrophy in neurodegeneration -a meta-analysis. J. Neurol. Neurosurg. Psychiatry.

[bib0043] Gibbard C.R., Ren J., Seunarine K.K., Clayden J.D., Skuse D.H., Clark C.A. (2013). White matter microstructure correlates with autism trait severity in a combined clinical-control sample of high-functioning adults. Neuroimage Clin..

[bib0044] Gibbard C.R., Ren J., Skuse D.H., Clayden J.D., Clark C.A. (2017). Structural connectivity of the amygdala in young adults with autism spectrum disorder. Hum. Brain Mapp..

[bib0045] Golkov V., Dosovitskiy A., Sperl J.I., Menzel M.I., Czisch M., Samann P., Brox T., Cremers D. (2016). Q-space deep learning: twelve-fold shorter and model-free diffusion MRI scans. IEEE Trans. Med. Imaging.

[bib0046] Goodfellow I., Bengio Y., Courville A. (2016). Deep Learning.

[bib0047] Grebenkov D.S. (2010). Use, misuse, and abuse of apparent diffusion coefficients. Concepts Magn. Reson..

[bib0048] Gyori N.G., Clark C.A., Dragonu I., Alexander D.C., Kaden E. (2019). In-vivo neural soma imaging using b-tensor encoding and deep learning. Proceedings of the ISMRM.

[bib0049] Henriques R.N., Jespersen S.N., Shemesh N. (2020). Correlation tensor magnetic resonance imaging. Neuroimage.

[bib0050] Hill I., Palombo M., Santin M., Branzoli F., Philippe A., Wassermann D., Aigrot M., Stankoff B., Baron-Van Evercooren A., Felfi M., Langui D., Zhang H., Lehericy S., Patiet A., Alexander D.C., Ciccarelli O., Drobnjak I. (2021). Machine learning based white matter models with permeability: an experimental study in cuprizone treater in-vivo mouse model of axonal demyelination.

[bib0028] Hygino da Cruz L.C.J., Batista R.R., Domingues R.C., Barkhof F. (2011). Diffusion magnetic resonance imaging in multiple sclerosis. Neuroimaging Clin. N. Am..

[bib0051] Innocenti G.M., Caminiti R. (2016). Axon diameter relates to synaptic bouton size: structural properties define computationally different types of cortical connections in primates. Brain Struct. Funct..

[bib0052] Jelescu I.O., Budde M.D. (2017). Design and validation of diffusion MRI models of white matter. Front. Phys..

[bib0053] Jelescu I.O., Veraart J., Fieremans E., Novikov D.S. (2016). Degeneracy in model parameter estimation for multi-compartment diffusion in neuronal tissue. NMR Biomed..

[bib0054] Jernigan T.L., Baare W.F.C., Stiles J., Skak Madsen K. (2011). Postnatal brain development: structural imaging of dynamic neurodevelopmental processes. Prog. Brain Res..

[bib0055] Jespersen S.N., Kroenke C.D., Ostergaard L., Ackermann J.J., Yablonskiy D.A. (2007). Modeling dendrite density from magnetic resonance diffusion measurements. Neuroimage.

[bib0056] Jespersen S.N., Lundell H., Sonderby C.K., Dyrby T.B. (2013). Orientationally invariant metrics of apparent compartment eccentricity from double pulsed field gradient diffusion experiments. NMR Biomed..

[bib0057] Jespersen S.N., Olesen J.L., Ianus A., Shemesh N. (2019). Effects of nonGaussian diffusion on “isotropic diffusion” measurements: an ex-vivo microimaging and simulation study. J. Magn. Reson..

[bib0058] Jiang G., Yin X., Li C., Li L., Zhao L., Evans A.C., Jiang T., Wu J., Wang J. (2015). The plasticity of brain gray matter and white matter following lower limb amputation. Neural Plast..

[bib0059] Johansen-Berg H., Behrens T.E.J. (2009). Diffusion MRI: From Quantitative Measurement to in-vivo Neuroanatomy.

[bib0060] Johns P. (2014). Clinical Neuroscience: An Illustrated Colour Text.

[bib0061] Jones D.K. (2011). Diffusion MRI: Theory, Methods, and Applications.

[bib0062] Jones D.K., Simmons A., Williams S.C.R., Horsfield M.A. (1999). Non-invasice assessment of axonal fiber connectivity in the human brain via diffusion tensor MRI. Magn. Reson. Med..

[bib0063] Kaden E., Kelm N.D., Carson R.P., Does M.D., Alexander D.C. (2016). Multi-compartment microscopic diffusion imaging. Neuroimage.

[bib0064] Kaden E., Knosche T.R., Anwander A. (2007). Parametric spherical deconvolution: inferring anatomical connectivity using diffusion mr imaging. Neuroimage.

[bib0065] Kaden E., Kruggel F., Alexander D.C. (2016). Quantitative mapping of the per-axon diffusion coefficients in brain white matter. Magn. Reson. Med..

[bib0066] Kantarci K., Murray M.E., Schwarz C.G., Reid R.I., Przybelski S.A., Lesnick T., Zuk S.M., Raman M.R., Senjem M.L., Gunter J.L., Boeve B.F., Knopman D.S., Parisi J.E., Petersen R.C., Jack C.R.J., Dickson D.W. (2017). White-matter integrity on DTI and the pathologic staging of Alzheimer’s disease. Neurobiol. Aging.

[bib0067] Kellner E., Dhital B., Kiselev V.G., Reisert M. (2016). Gibbs-ringing artifact removal based on local subvoxel-shifts. Magn. Reson. Med..

[bib0068] Kiselev V.G., Jones D.K. (2010). The cumulant expansion: an overarching mathematical framework for understanding diffusion NMR. Diffusion MRI: Theory, Methods and Applications.

[bib0069] Koch M.A., Finsterbusch J. (2008). Compartment size estimation with double wave vector diffusion-weighted imaging. Magn. Reson. Med..

[bib0070] Komlosh M.E., Horkay F., Freidlin R.Z., Nevo U., Assaf Y., Basser P.J. (2007). Detection of microscopic anisotropy in gray matter and in a novel tissue phantom using double pulsed gradient spin echo mr. J. Magn. Reson..

[bib0071] Komlosh M.E., Ozarslan E., Lizak M.J., Horkay F., Schram V., Shemesh N., Cohen Y., Basser P.J. (2011). Pore diameter mapping using double pulsed-field gradient MRI and its validation using a novel glass capillary array phantom. J. Magn. Reson..

[bib0072] Lampinen B., Szczepankiewicz F., Martensson J., van Westen D., Sundgren P.C., Nilsson M. (2017). Neurite density imaging versus imaging of microscopic anisotropy in diffusion MRI: a model comparison using spherical tensor encoding. Neuroimage.

[bib0073] Lampinen B., Szczepankiewicz F., Noven M., van Westen D., Hansson O., Englund E., Martensson J., Westin C.-F., Nilsson M. (2019). Searching for the neurite density with diffusion MRI: challenges for biophysical modelling. Hum. Brain Mapp..

[bib0074] Lasic S., Szczepankiewicz F., Eriksson S., Nilsson M., Topgaard D. (2014). Microanisotropy imaging: quantification of microscopic diffusion anisotropy and orientational order parameter by diffusion MRI with magic-angle spinning of the q-vector. Front. Phys..

[bib0075] Laule C., Leung E., Li D.K.B., Traboulsee A.L., Paty D.W., MacKay A.L., Moore G.R.W. (2006). Myelin water imaging in multiple sclerosis: quantitative correlations with histopathology. Mult. Scler..

[bib0076] Le Bihan D., Breton E., Lallemand D., Grenier P., Cabanis E., Laval-Jeantet M. (1986). MR imaging of intravoxel incoherent motions: application to diffusion and perfusion in neurologic disorders. Radiology.

[bib0077] Lundell H., Nilsson M., Dyrby T.B., Parker G.J.M., Cristinacce H., Zhou F.-L., Topgaard D., Lasic S. (2019). Multidimensional diffusion MRI with spectrally modulated gradients reveals unprecedented microstructural detail. Sci. Rep..

[bib0078] Lutsep H.L., Albers G.W., de Crespigny A., Kamat G.N., Marks M.P., Moseley M.E. (1997). Clinical utility of diffusion-weighted magnetic resonance imaging in the assessment of ischemic stroke. Ann. Neurol..

[bib0079] MacKay A., Whittall K., Adler J., Li D., Paty D., Graeb D. (1994). In vivo visualization of myelin water in brain by magnetic resonance. Magn. Reson. Med..

[bib0080] McColgan P., Seunarine K.K., Gregory S., Razi A., Papoutse M., Long J.D., Mills J.A., Johnson E., Durr A., Roos R.A.C., Leavitt B.R., Stout J.C., Scahill R.I., Clark C.A., Rees G., Tabrizi S.J., Investigators T.T.H. (2017). Topological length of white matter connections predicts their rate of atrophy in premanifest Huntington’s disease. J. Clin. Invest..

[bib0081] McKinnon E.T., Jensen J.H. (2019). Measuring intra-axonal t2 in white matter with direction-averaged diffusion MRI. Magn. Reson. Med..

[bib0082] McKinnon E.T., Jensen J.H., Glenn G.R., Helpern J.A. (2017). Dependence on b-value of the direction-averaged diffusion-weighted imaging signal in brain. Magn. Reson. Imaging.

[bib0083] Minatogawa-Chang T.M., Schaufelberger M.S., Ayres A.M., Duran F.L.S., Gutt E.K., Murray R.M., Rushe T.M., McGuire P.K., Menezes P.R., Scazufca M., Busatto G.F. (2009). Cognitive performance is related to cortical grey matter volumes in early stages of schizophrenia: a population-based study of the first-episode psychosis. Schizophr. Res..

[bib0084] Mitra P.P. (1995). Multiple wave-vector extensions of the NMR pulsed-field-gradient spin-echo diffusion measurement. Phys. Rev. B.

[bib0085] Mori S., van Zjil P.C.M. (1995). Diffusion weighting by the trace of the diffusion tensor within a single scan. Magn. Reson. Med..

[bib0086] Nedjati-Gilani G.L., Schneider T., Hall M.C., Cawley N., Hill I., Ciccarelli O., Drobnjak I., Gandini Wheeler-Kingshott C.A.M., Alexander D.C. (2017). Machine learning based compartment models with permeability for white matter microstructure imaging. Neuroimage.

[bib0087] Nedjati-Gilani, G. L., Schneider, T., Hall, M. C., Wheeler-Kingshott, C. A. M., Alexander, D. C., 2014. Machine learning based compartment models with permeability for white matter microstructure imaging. 10.1007/978-3-319-10443-0_33 25320807

[bib0088] Niendorf T., Dijkhuizen R.M., Norris D.G., van Lookeren Campagne M., Nicolay K. (1996). Biexponential diffusion attenuation in various states of brain tissue: implications for diffusion-weighted imaging. Magn. Reson. Med..

[bib0089] Nilsson M., Alerstam E., Wirestam R., Stahlberg F., Brockstedt S., Latt J. (2010). Evaluating the accuracy and precision of a two-compartment Karger model using monte carlo simulations. J. Magn. Reson..

[bib0090] Novak M.J., Seunarine K.K., Gibbard C.R., Hobbs N.Z., Scahill R.I., Clark C.A., Tabizi S.J. (2014). White matter integrity in premanifest and early huntington’s disease is related to caudate loss and disease progression. Cortex.

[bib0091] Novikov D.S., Veraart J., Jelescu I.O., Fieremans E. (2018). Rotationally-invariant mapping of scalar and orientational metrics of neuronal microstructure with diffusion MRI. Neuroimage.

[bib0092] Ozarslan E. (2009). Compartment shape anisotropy (CSA) revealed by double pulsed field gradient MR. J. Magn. Reson..

[bib0093] Ozarslan E., Basser P.J. (2008). Microscopic anisotropy revealed by NMR double pulsed field gradient experiments with arbitrary timing parameters. J. Chem. Phys..

[bib0094] Ozarslan E., Yolcu C., Herberthson M., Knutsson H., Wetin C.-F. (2018). Influence of the size and curvedness of neural projections on the orientationally averaged diffusion mr signal. Front. Phys..

[bib0095] Palombo M., Ianus A., Guerreri M., Nunes D., Alexander D.C., Shemesh N., Zhang H. (2020). Sandi: a compartment-based model for non-invasive apparent soma and neurite imaging by diffusion MRI. Neuroimage.

[bib0096] Palombo M., Ianus A., Guerreri M., Nunes D., Alexander D.C., Shemesh N., Zhang H. (2021). Corrigendum to “Sandi: a compartment-based model for non-invasive apparent soma and neurite imaging by diffusion mri” [neuroimage 215 (2020), 116835]. Neuroimage.

[bib0097] Palombo M., Nunes D., Alexander D.C., Zhang H., Shemesh N. (2019). Histological validation of the brain cell body imaging with diffusion MRI at ultrahigh field. Proceedings of the ISMRM.

[bib0098] Palombo M., Shemesh N., Ianus A., Alexander D.C., Zhang H. (2018). A compartment based model for non-invasive cell body imaging by diffusion MRI. Proceedings of the ISMRM.

[bib0099] Panagiotaki E., Schneider T., Siow B., Hall M.G., Lythgoe M.F., Alexander D.C. (2012). Compartment models of the diffusion mr signal in brain white matter: a taxonomy and comparison. Neuroimage.

[bib0100] Perani S., Tierney T.M., Centeno M., Shamshiri E.A., Yaakub S.N., O’Muicheartaigh J., Carmichael D.W., Richardson M.P. (2018). Thalamic volume reducion in drug-naive patients with new-onset genetic generalized epilepsy. Epilepsia.

[bib0101] Reisert M., Kiselev V.G., Dhital B. (2019). A unique analytical solution of the white matter standard model using linear and planar encodings. Magn. Reson. Med..

[bib0102] Robert C.P. (2007). The Bayesian Choice.

[bib0103] Schmierer K.S., Wheeler-Kingshott C.A.M., Boulby P.A., Scaravilli F., Altmann D.R., Barker G.J., Tofts P.S., Miller D.H. (2007). Diffusion tensor imaging of post mortem multiple sclerosis brain. Neuroimage.

[bib0104] Schouten T.M., Koini M., Vos F., Seiler S., Rooij M., Lechner A., Schmidt R., Heuvel M.V.D., Grond J.V., Rombouts S.A.R.B. (2017). Individual classification of alzheimer’s disease with diffusion magnetic resonance imaging. Neuroimage.

[bib0105] Setsompop K., Gagoski B.A., Polimeni J.R., Witzel T., Wedeen V.J., Wald L.L. (2012). Blipped-controlled aliasing in parallel imaging (blipped-caipi) for simultaneous multi-slice EPI with reduced g-factor penalty. Magn. Reson. Med..

[bib0106] Shemesh N., Cohen Y. (2011). Microscopic and compartment shape anisotropies in gray and white matter revealed by angular bipolar double-PFG MR. Magn. Reson. Med..

[bib0107] Shemesh N., Ozarslan E., Basser P.J., Cohen Y. (2010). Detecting diffusion-diffraction patterns in size distribution phantoms using double-pulsed field gradient NMR: theory and experiments. J. Chem. Phys..

[bib0108] Shemesh N., Ozarslan E., Kolmosh M.E., Basser P.J., Cohen Y. (2010). From single-pulsed field gradient to double-pulsed field gradient mr: gleaning new microstructural information and developing new forms of contrast in MRI. NMR Biomed..

[bib0109] Shepherd G.M. (1998). The Synaptic Organization of the Brain.

[bib0110] Sjolund J., Szczepankiewicz F., Nilsson M., Topgaard D., Westin C.-F., Knutsson H. (2015). Constrained optimization of gradient waveforms for generalized diffusion encoding. J. Magn. Reson..

[bib0111] Smith S.M. (2002). Fast robust automated brain extraction. Hum. Brain Mapp..

[bib0112] Smith S.M., Jenkinson M., Woolrich M.W., Beckmann C.F., Behrens T.E.J., Johansen-Berg H., Bannister P.R., De Luca M., Drobnjak I., Flitney D.E., Niazy R.K., Saunders J., Vickers J., Zhang Y., De Stefano N., Michael Brady J., Matthews P.M. (2004). Advances in functional and structural mr image analysis and implementation as FSL. Neuroimage.

[bib0113] Spijkerman J.M., Petersen E.T., Hendrikse J., Luijten P., Zwanenburg J.J.M. (2018). T2 mapping of cerebrospinal fluid: 3 t versus 7 t. Magn. Reson. Mater. Phys. Biol. Med..

[bib0114] Stanisz G.J., Szafer A., Wright G.A., Henkelman R.M. (1997). An analytical model of restricted diffusion in bovine optic nerve. Magn. Reson. Med..

[bib0115] Szczepankiewicz F., Hoge S., Westin C.-F. (2019). Linear, planar and spherical tensor-valued diffusion MRI data by free waveform encoding in healthy brain, water, oil and liquid crystals. Data Brief.

[bib0116] Szczepankiewicz F., Lasic S., van Westen D., Sundgren P.D., Englund E., Westin C.-F., Stahlberg F., Latt J., Topgaard D., Nilsson M. (2015). Quantification of microscopic diffusion anisotropy disentangles effects of orientation dispersion from microstructure: applications in volunteers and in brain tumors. Neuroimage.

[bib0117] Szczepankiewicz F., Sjolund J., Stahlberg F., Latt J., Nilsson M. (2019). Tensor-valued diffusion encoding for diffusional variance decomposition (divide): technical feasibility in clinical MRI systems. PLoS ONE.

[bib0118] Szczepankiewicz F., van Westen D., Englund E., Westin C.-F., Stahlberg F., Latt J., Sundgren P.D., Nilsson M. (2016). The link between diffusion MRI and tumor heterogeneity: mapping cell eccentricity and density by diffusional variance decomposition (divide). Neuroimage.

[bib0119] Szczepankiewicz F., Westin C.-F., Nilsson M. (2019). Maxwell-compensated design of asymmetric gradient waveforms for tensor-valued diffusion encoding. Magn. Reson. Med..

[bib0120] Tang Y.-Y., Lu Q., Fan M., Yang Y., Pasner M.I. (2012). Mechanisms of white matter changes induced by meditation. Proc. Natl. Acad. Sci. U.S.A..

[bib0121] Tax C.M.W., Szczepankiewicz F., Nilsson M., Jones D.K. (2020). The dot-compartment revealed? diffusion MRI with ultra-strong gradients and spherical tensor encoding in the living human brain. Neuroimage.

[bib0122] Topgaard D. (2017). Multidimensional diffusion MRI. J. Magn. Reson..

[bib0123] Tournier J.D., Calamante F., Connelly A. (2010). Improved probabilistic streamlines tractography by 2nd order integration over fibre orientation distributions. Proceedings of the 18th Annual Meeting of the ISMRM.

[bib0124] Tournier J.D., Smith R.E., Raffelt D.A., Tabbara R., Dhollander T., Pietsch M., Chistiaens D., Jeurissen B., Yeh C., Connelly A. (2019). Mrtrix3: a fast, flexible and open software framework for medical image processing and visualisation. Neuroimage.

[bib0125] Travers B.G., Adluru N., Ennis C., Tromp D.P.M., Destiche D., Doran S., Bigler E.D., Lange N., Lainhart J.E., Alexander A.L. (2012). Diffusion tensor imaging in autism spectrum disorder: a review. Autism Res..

[bib0126] Tyborowska A., Volman I., Niermann H.C.M., Pouwels L., Smeekens S., Cillessen A.H.N., Toni I., Roelofs K. (2018). Early-life and pubertal stress differentially modulate grey matter development in human adolescents. Sci. Rep..

[bib0127] Veraart J., Novikov D.S., Fieremans E. (2018). Te dependent diffusion imaging (teddi) distinguishes between compartmental T2 relaxation times. Neuroimage.

[bib0128] Veraart J., Nunes D., Rudrapatna U., Fieremans E., Jones D.K., Novikov D.S., Shemesh N. (2020). Noninvasive quantification of axon radii using diffusion MRI. Elife.

[bib0129] Vercellino M., Masera S., Lorenzatti M., Condello C., Merola A., Mattioda A., Tribolo A., Capello E., Mancardi G.L., Mutani R., Giordana M.T., Cavalla P. (2009). Demyelination, inflammation and neurodegeneration in multiple sclerosis deep gray matter. J. Neuropathol. Exp. Neurol..

[bib0130] Warach S., Chien D., Li W., Ronthal M., Edelman R.R. (1992). Fast magnetic resonance diffusion-weighted imaging of acute human stroke. Neurology.

[bib0131] Weber T., Ziener C.H., Kampf T., Herold V., Bauer W.R., Jakob P.M. (2009). Measurement of apparent cell radii using a multiple wave vector diffusion experiment. Magn. Reson. Med..

[bib0132] Werring D.J., Clark C.A., Barker G.J., Thompson A.J., Miller D.H. (1999). Diffusion tensor imaging of lesions and normal appearing white matter in multiple sclerosis. Neurology.

[bib0133] West K.L., Kelm N.D., Carson R.P., Alexander D.C., Gochberg D.F., Does M.D. (2018). Experimental studies of g-ratio MRI in ex vivo mouse brain. Neuroimage.

[bib0134] Westin C.-F., Knutsson H., Paternak O., Szczepankiewicz F., Ozarslan E., van Westen D., Mattisson C., Bogren M., O’Donnell L.J., Kubicki M., Topgaard D., Nilsson M. (2016). Q-space trajectory imaging for multidimensional diffusion MRI of the human brain. Neuroimage.

[bib0135] Westin C.-F., Szczepankiewicz F., Paternak O., Ozarslan E., Topgaard D., Knutsson H., Nilsson M. (2014). Measurement tensors in diffusion MRI: generalizing the concept of diffusion encoding. Proceedings of the Medical Image Computing and Computer-Assisted Intervention: MICCAI, International Conference on Medical Image Computing and Computer-Assisted Intervention.

[bib0136] Weston P.S.J., Simpson I.J.A., Ryan N.S., Ourselin S., Fox N.S. (2015). Diffusion imaging changes in grey matter in Alzheimer’s disease: a potential marker of early neurodegeneration. Alzheimer’s Res. Ther..

[bib0137] Wierda K.D.B., Sorensen J.B. (2014). Innervation by a GABAergic neuron depresses spontaneous release in glutamatergic neurons and unveils the clamping phenotype of synaptotagmin-1. J. Neurosci..

[bib0138] Wong E.C., Cox R.W., Song A.W. (1995). Optimized isotropic diffusion weighting. Magn. Reson. Med..

[bib0139] Young J.M., Powell T.L., Morgan B.R., Card D., Lee W., Smith M.L., Sled J.G., Taylor M.J. (2015). Deep grey matter growth predicts neurodevelepmental outcomes in very preterm children. Neuroimage.

[bib0140] Zatorre R.J., Fields R.D., Johansen-Berg H. (2012). Plasticity in gray and white: neuroimaging changes in brain structure during learning. Nat. Neurosci..

[bib0141] Zhang H., Schneider T., Wheeler-Kingshott C.A., Alexander D.C. (2012). Noddi: practical in vivo neurite orientation dispersion and density imaging of the human brain. Neuroimage.

[bib0142] Zimmerman M.E., Brickman A.M., Paul R.H., Grieve S.M., Tate D.F., Gunstad J., Cohen R.A., Aloia M.S., Williams L.M., Clark C.R., Whitford T.J., Gordon E. (2006). The relationship between frontal gray matter volume and cognition varies across the healthy adult lifespan. Am. J. Geriatr. Psychiatry.

