# Supplementary material for: On the potential for mapping apparent neural soma density via a clinically viable diffusion MRI protocol
Source: Neuroimage. 2021 Oct 1;239:118303. doi: 10.1016/j.neuroimage.2021.118303 (PMC8363942; doi:10.1016/j.neuroimage.2021.118303)
Supplement: Supplementary Data S1 — Supplementary Raw Research Data. This is open data under the CC BY license http://creativecommons.org/licenses/by/4.0/ [file mmc1.pdf]

Supplementary Material

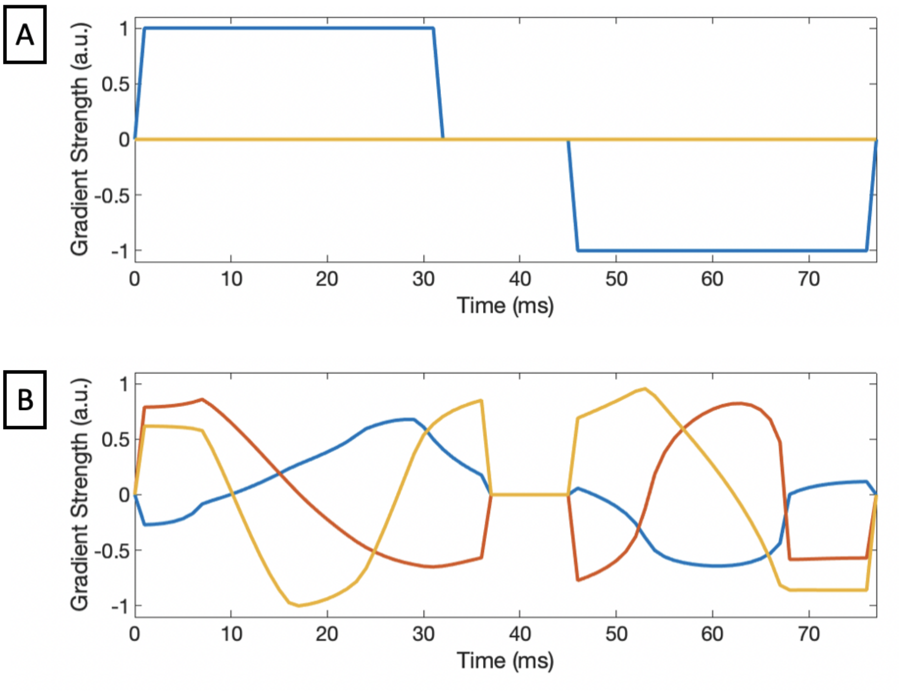

Figure S1: (A) LTE and (B) STE waveforms used in this study.

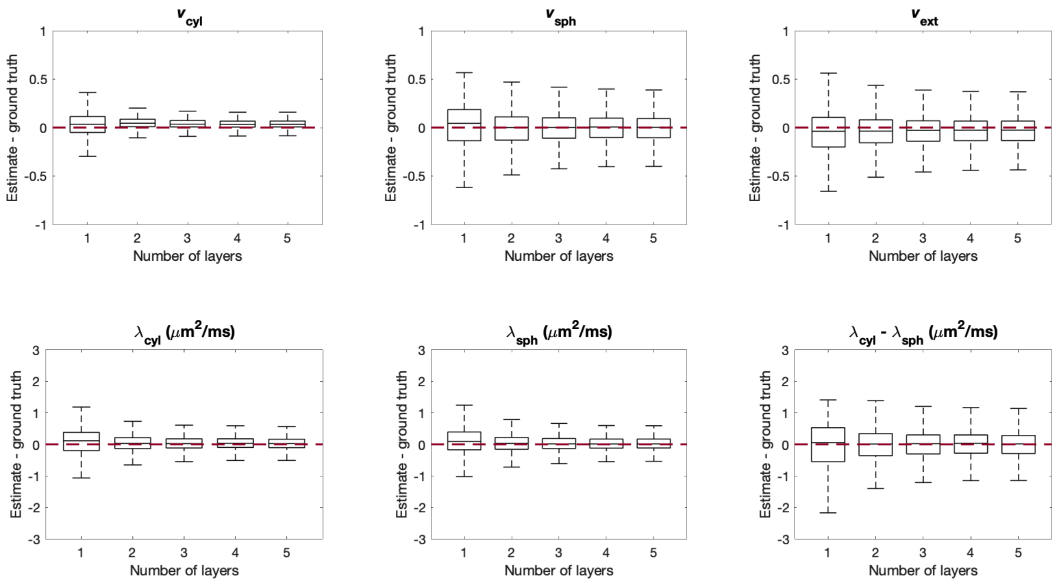

Figure S2: Box plots showing the estimation errors of all parameters for different numbers of layers in the neural network. Parameters were estimated for the whole range of biophysically plausible values with added noise of SNR=25. The figure shows that the parameter estimation is reasonably accurate with three network layers and does not significantly improve with more layers.

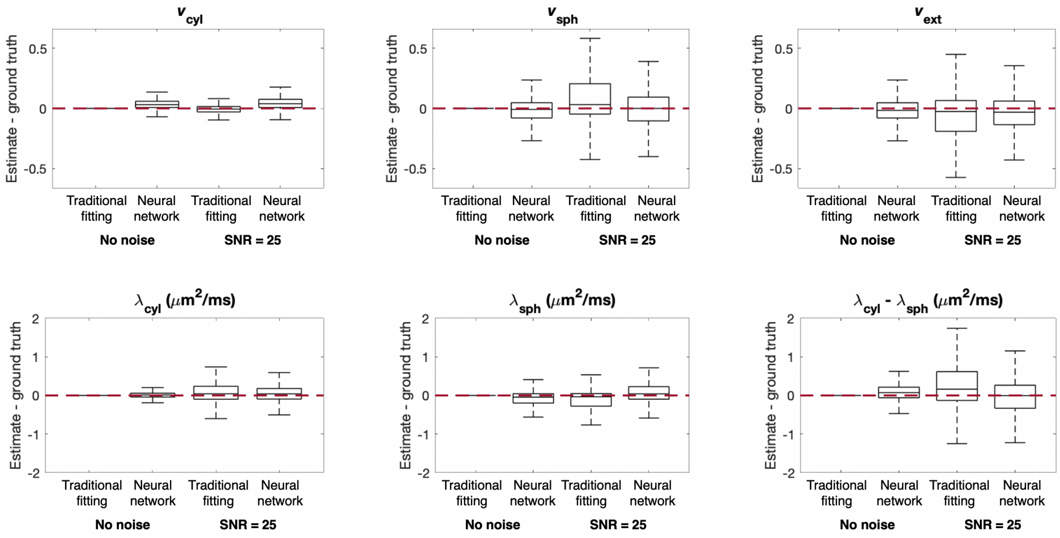

Figure S3: The difference between parameter estimates and ground truth values using simulated data. The plots compare parameter estimates using traditional model fitting and the artificial neural network for the conditions of no noise and noise with SNR = 25. The red line indicates no difference between parameter estimates and ground truth values. When no noise is added to the test data, traditional model fitting estimates parameters with high accuracy and the corresponding box plot has no perceivable width. The errors for parameter estimation are higher for the neural network in the no noise condition, as a three-layer network has a limited model capacity. This error can be reduced by adding more network layers (see also Figure S2).

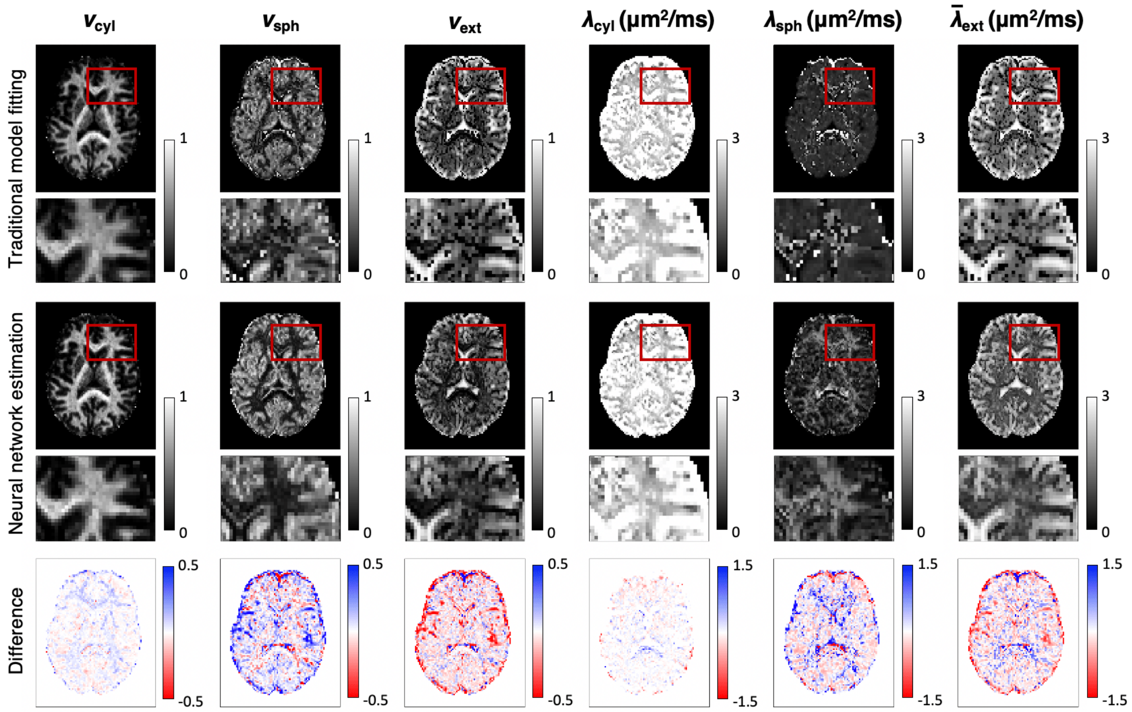

Figure S4: Maps of apparent parameter estimates from both traditional model fitting and the artificial neural network. The last row shows the difference between traditional model fitting estimates and estimates using the artificial neural network. The maps look similar for both fitting techniques. Zoomed plots demonstrate that the maps from traditional model fitting tend to be noisier. For example, the estimates of spherical compartment diffusivity are noisier in white matter for traditional model fitting. This is likely because the volume fraction of spherical compartments is very low in this region, making the diffusivity of these cellular geometries more difficult to estimate. This shows that in regions of high uncertainty, parameter estimates from traditional model fitting appear noisy, whereas estimates from the neural network are smooth.

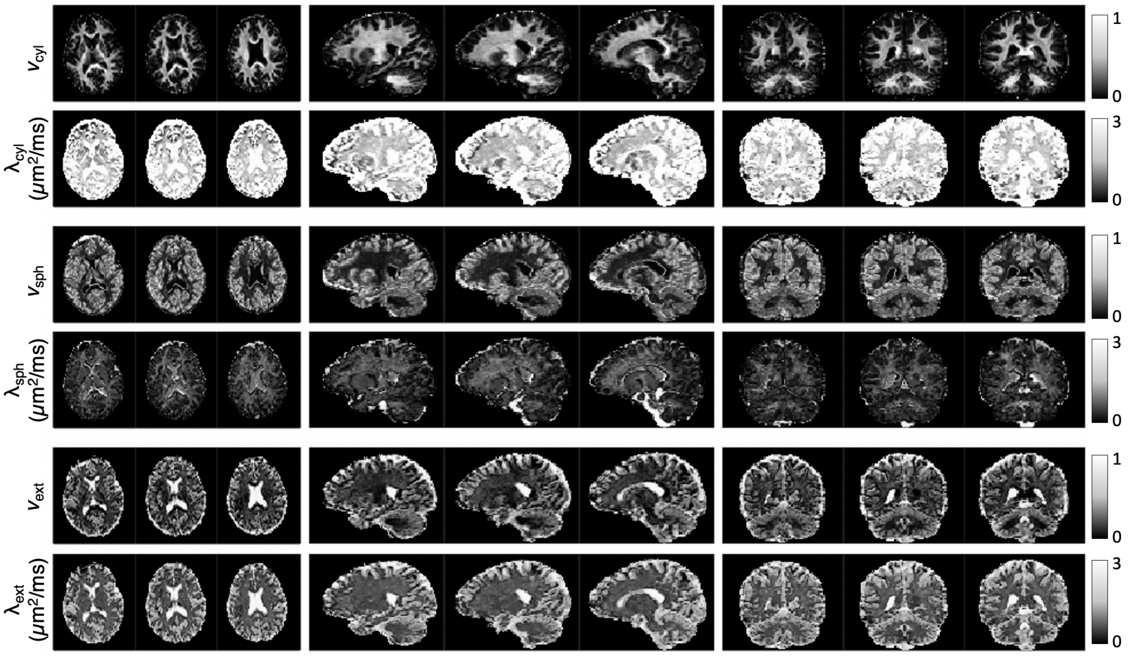

Figure S5: Apparent volume fraction and diffusivity of microscopic compartments in brain grey and white matter for subject 2.

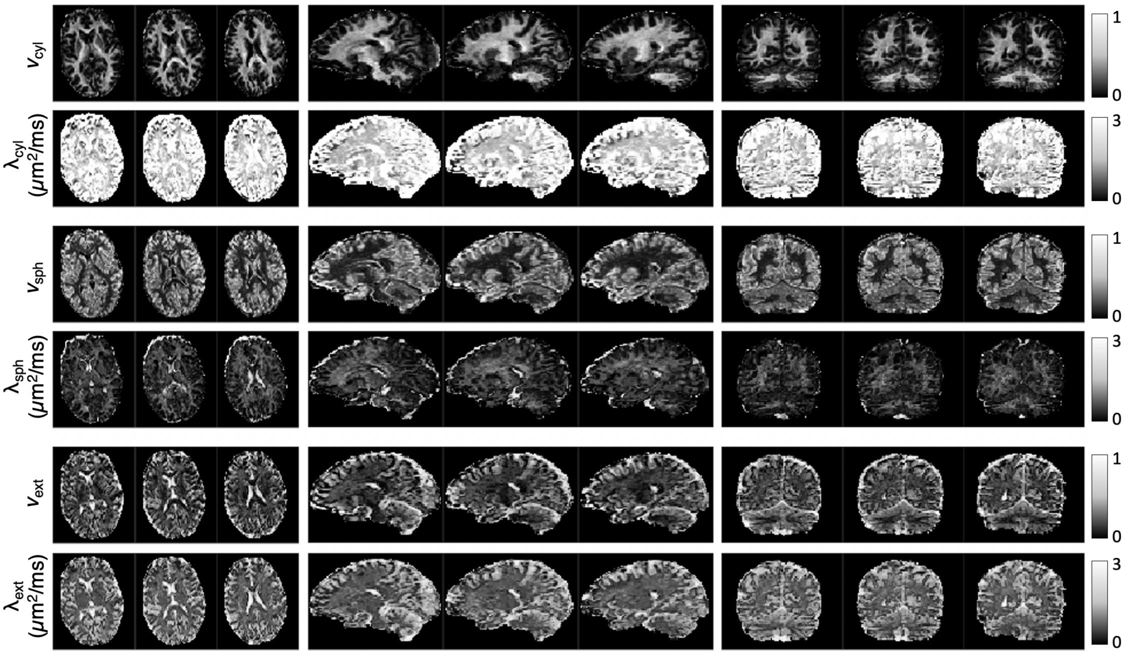

Figure S6: Apparent volume fraction and diffusivity of microscopic compartments in brain grey and white matter for subject 3.

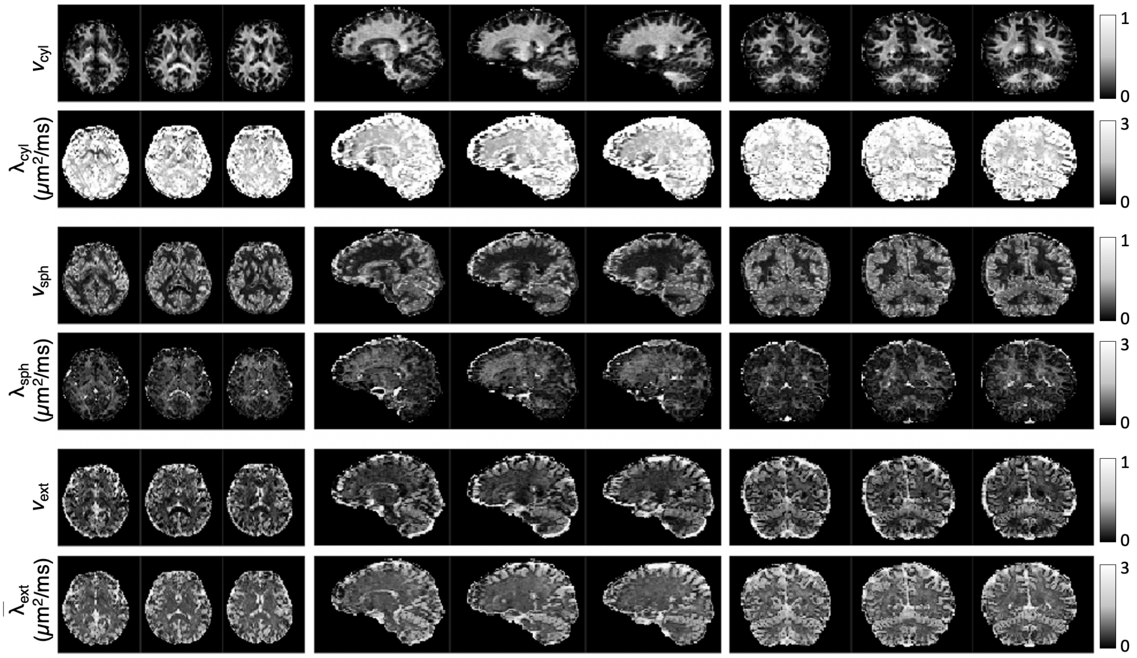

Figure S7: Apparent volume fraction and diffusivity of microscopic compartments in brain grey and white matter for subject 4.

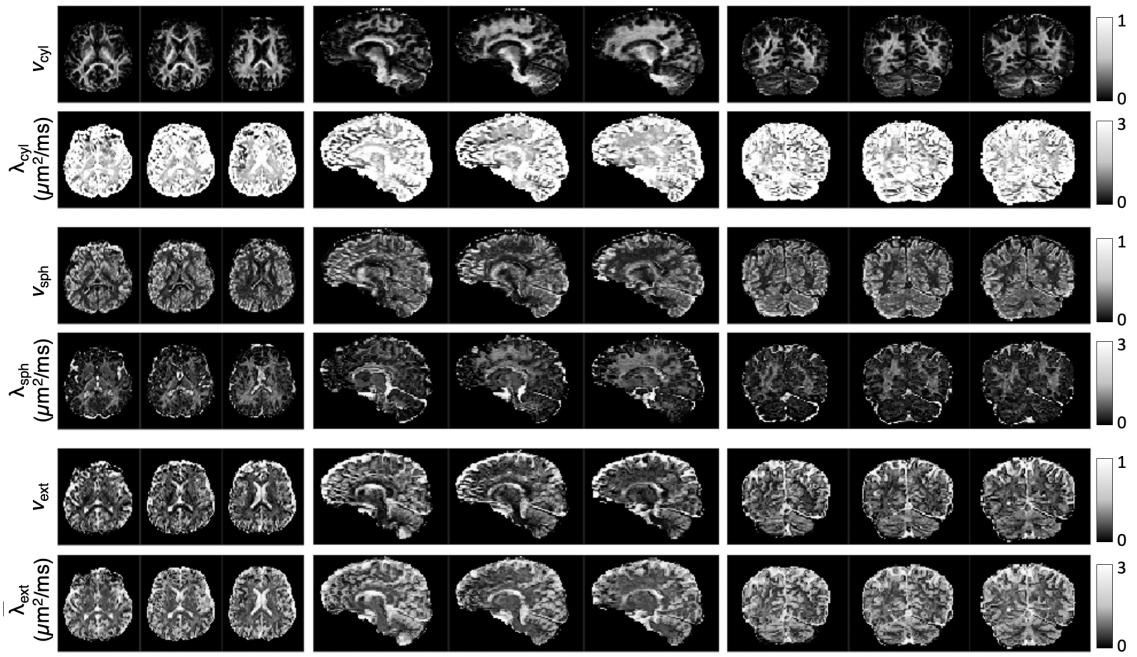

Figure S8: Apparent volume fraction and diffusivity of microscopic compartments in brain grey and white matter for subject 5.

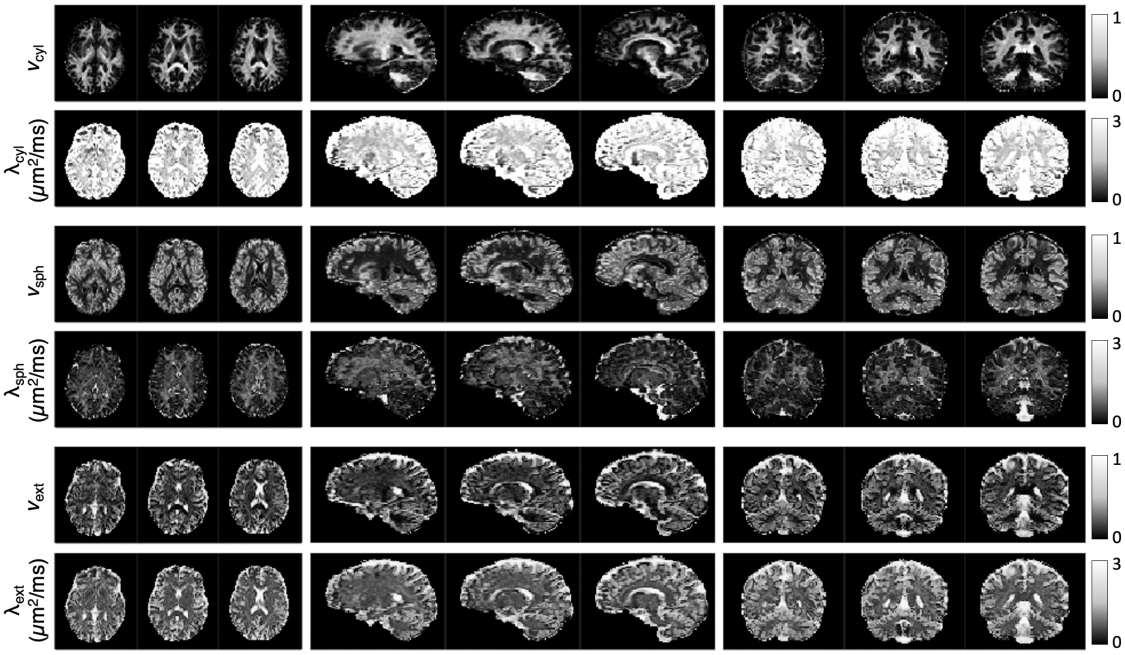

Figure S9: Apparent volume fraction and diffusivity of microscopic compartments in brain grey and white matter for subject 6.

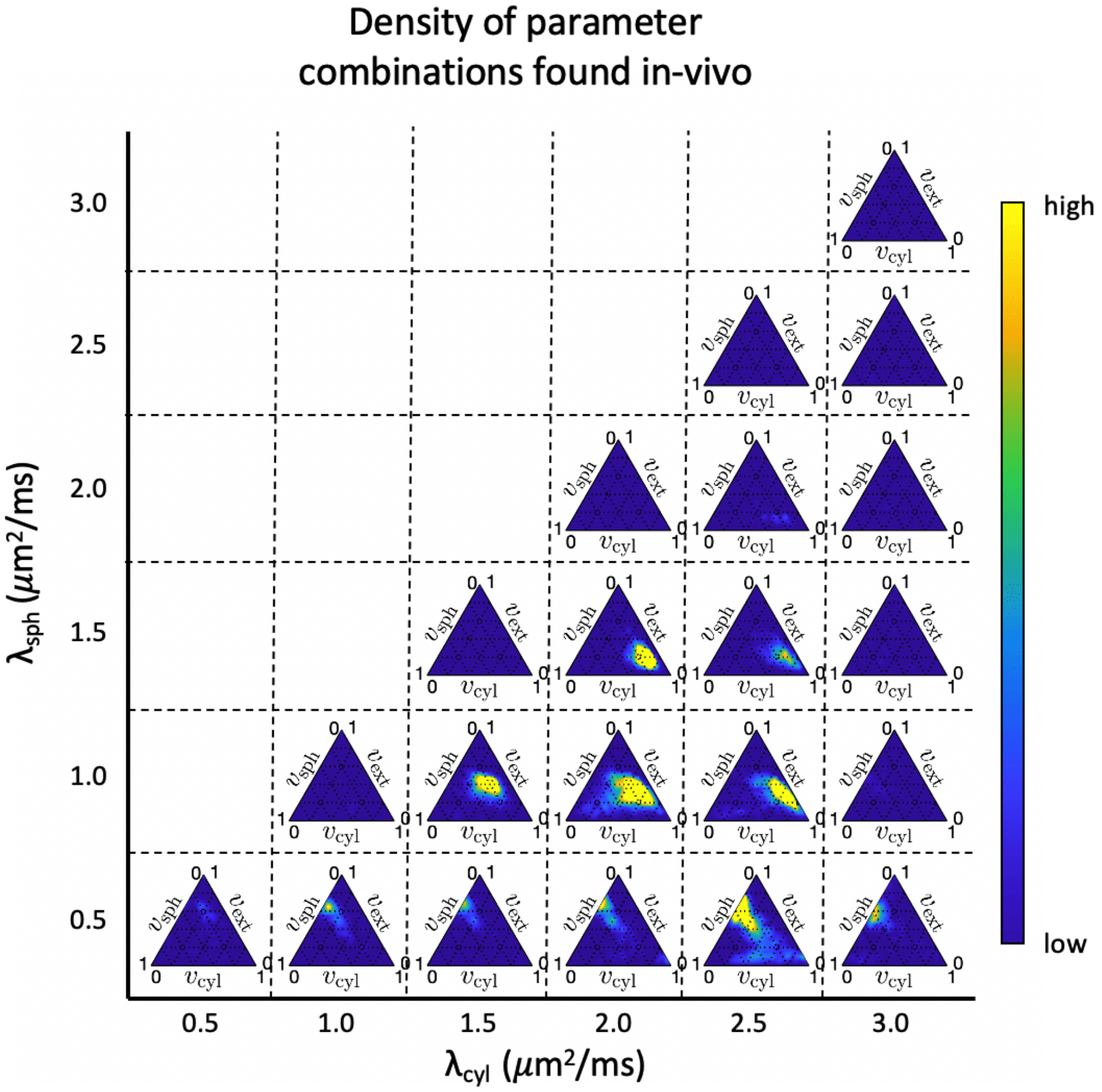

Figure S10: Density of parameter combinations found in white and grey matter regions of the six in-vivo data sets used in this work. Parameter combinations that are typically found appear yellow, whereas atypical parameter combinations appear blue, as shown by the colour bar.

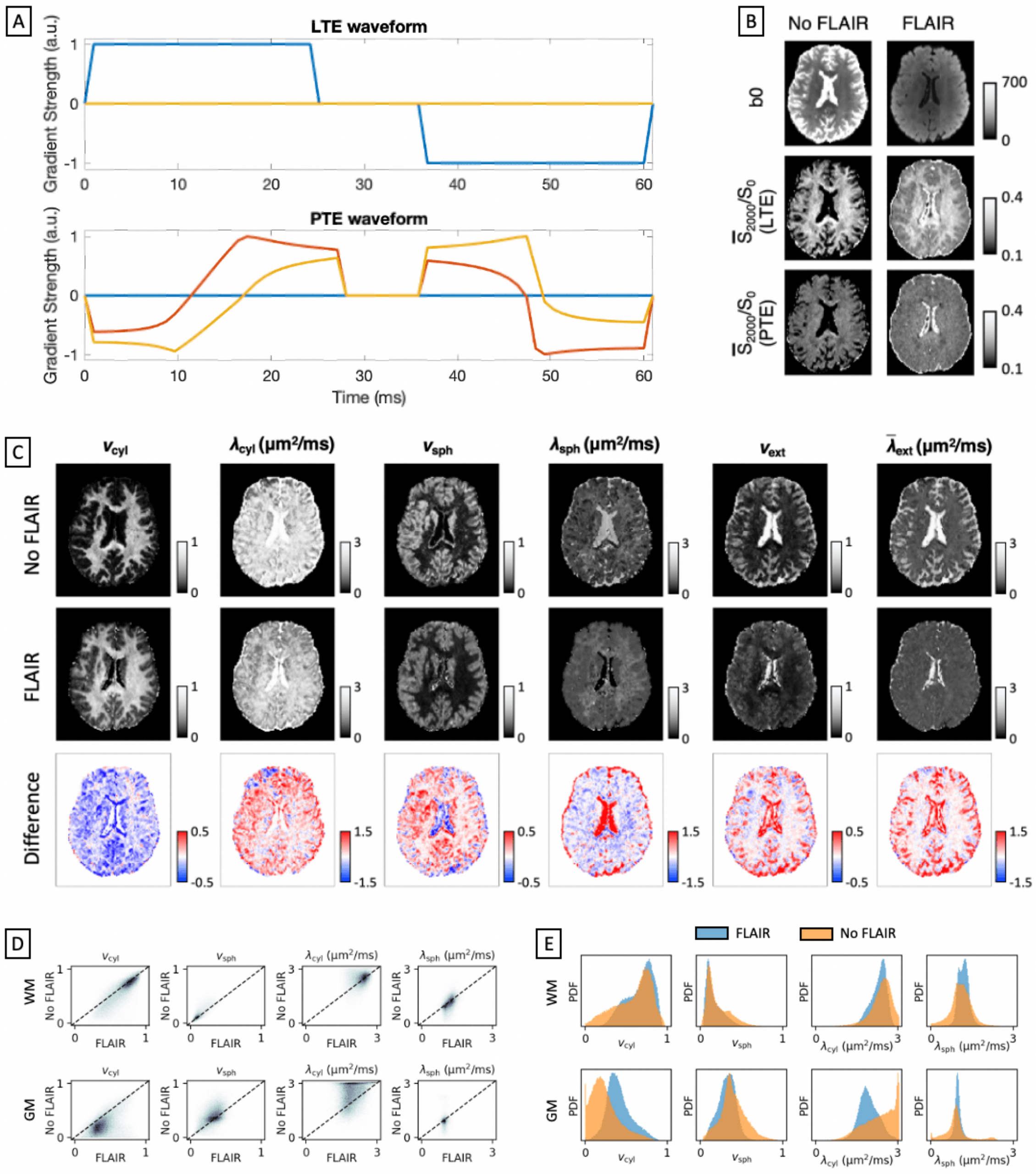

Figure S11: Complementary study with linear tensor encoding (LTE) and planar tensor encoding (PTE), fluid-attenuated inversion recovery (FLAIR) and high spatial resolution (1.5 mm isotropic voxels). Two data sets were acquired on the same volunteer: one with FLAIR to suppress the CSF signal, and another without FLAIR. Other measurement parameters were the same for the two data sets, including TE = 89 ms, TR = 23.4 s, LTE b-values of [1000, 2000, 3500, 5000] s/mm^2, PTE b-values of [500, 1000, 1500, 2000] s/mm^2 and 80 uniformly distributed gradient directions for both LTE and PTE. Panel (A) shows the B-tensor encoding waveforms and panel (B) shows the b0, and T2-normalised LTE and PTE maps for the two data sets with and without FLAIR. In panel (C), we compare the parameter estimates using the present model for the two data sets. In Panel (D) we show the correlation between the parameter estimates with and without FLAIR, whereas in Panel (E) we compare the distributions of the parameter estimates with and without FLAIR. For more details on methods and results, please refer to Gyori et al. (2020b).

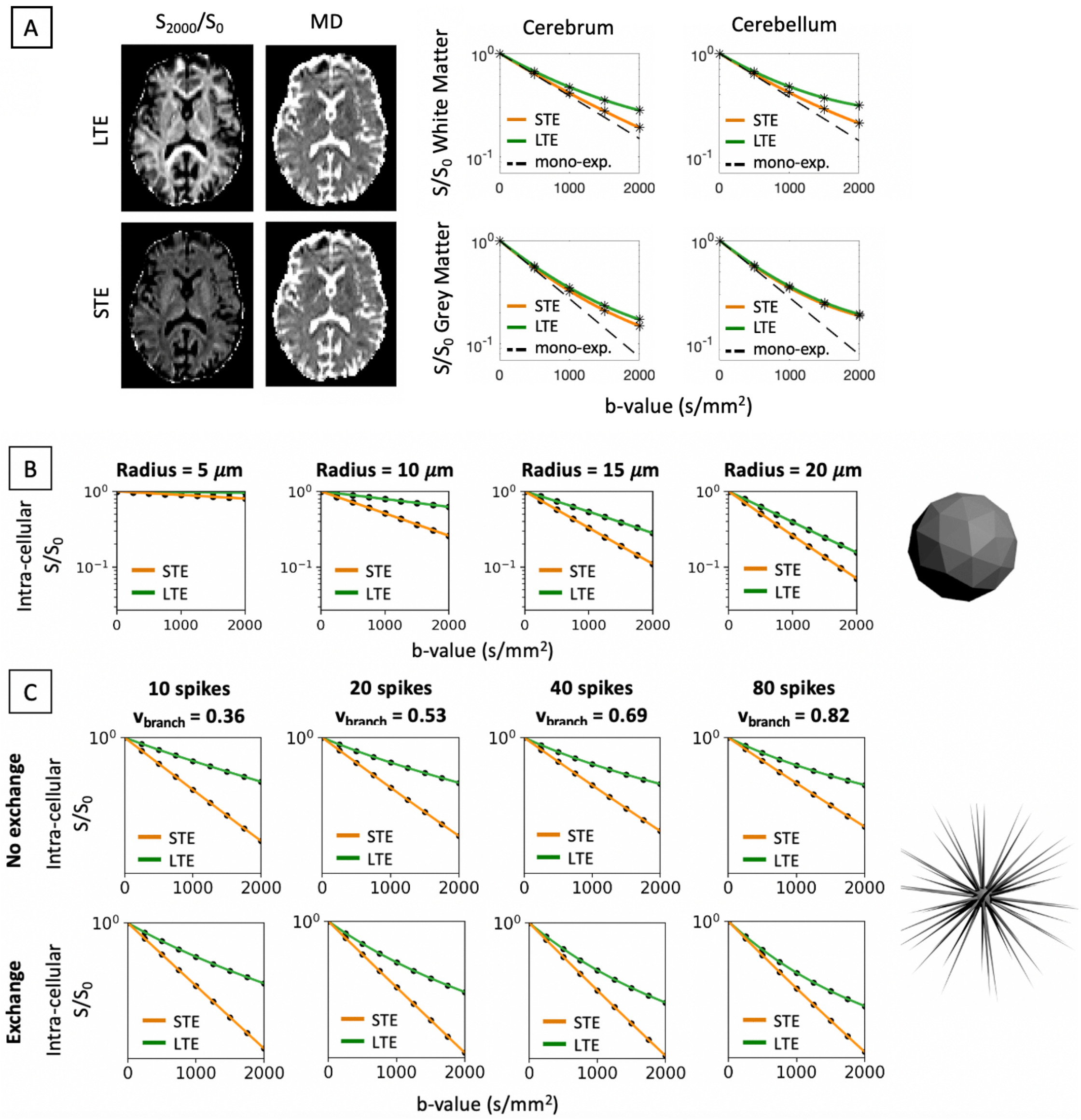

Figure S12: Panel (A): Mean diffusivity estimates from in-vivo data using LTE and STE. Signal decay curves show that LTE and STE measurements are similar at low b-values of approximately 500 s/mm^2, and hence the mean diffusivity that may be estimated is similar using both diffusion encoding waveforms. Panel (B): Monte-Carlo simulations of the diffusion signal within impermeable quasi-spherical compartments of different sizes, and the same LTE and STE waveforms as in the in-vivo measurements. While signal decay curves appear to be largely mono-exponential, LTE and STE diffusion signals are substantially different for the same b-value. In Panel (C), we used a quasi-spherical mesh of 10 μm with quasi-cylindrical protrusions as shown on the right. We simulated two conditions: firstly, when random walkers could not move between the spherical centre and the spikes, and secondly, when random walkers were free to move between the two. In the presence of water exchange between cell bodies and projections, the diffusion signal from LTE and STE are similar at low b-values. For higher b-values, the two curves are expected to diverge due to the presence of compartment anisotropy. The figure suggests that exchange processes between cell bodies and projections may be non-negligible in in-vivo tissue. For further details on methods and results, please refer to Gyori et al. (2020a).
